# Regulation of Budding Yeast CENP-A levels Prevents Misincorporation at Promoter Nucleosomes and Transcriptional Defects

**DOI:** 10.1371/journal.pgen.1005930

**Published:** 2016-03-16

**Authors:** Erica M. Hildebrand, Sue Biggins

**Affiliations:** 1 Howard Hughes Medical Institute, Division of Basic Sciences, Fred Hutchinson Cancer Research Center, Seattle, Washington, United States of America; 2 Molecular and Cellular Biology Program, University of Washington, Seattle, Washington, United States of America; Duke University, UNITED STATES

## Abstract

The exclusive localization of the histone H3 variant CENP-A to centromeres is essential for accurate chromosome segregation. Ubiquitin-mediated proteolysis helps to ensure that CENP-A does not mislocalize to euchromatin, which can lead to genomic instability. Consistent with this, overexpression of the budding yeast CENP-A^Cse4^ is lethal in cells lacking Psh1, the E3 ubiquitin ligase that targets CENP-A^Cse4^ for degradation. To identify additional mechanisms that prevent CENP-A^Cse4^ misincorporation and lethality, we analyzed the genome-wide mislocalization pattern of overexpressed CENP-A^Cse4^ in the presence and absence of Psh1 by chromatin immunoprecipitation followed by high throughput sequencing. We found that ectopic CENP-A^Cse4^ is enriched at promoters that contain histone H2A.Z^Htz1^ nucleosomes, but that H2A.Z^Htz1^ is not required for CENP-A^Cse4^ mislocalization. Instead, the INO80 complex, which removes H2A.Z^Htz1^ from nucleosomes, promotes the ectopic deposition of CENP-A^Cse4^. Transcriptional profiling revealed gene expression changes in the *psh1Δ* cells overexpressing CENP-A^Cse4^. The down-regulated genes are enriched for CENP-A^Cse4^ mislocalization to promoters, while the up-regulated genes correlate with those that are also transcriptionally up-regulated in an *htz1Δ* strain. Together, these data show that regulating centromeric nucleosome localization is not only critical for maintaining centromere function, but also for ensuring accurate promoter function and transcriptional regulation.

## Author Summary

Chromosomes carry the genetic material in cells. When cells divide, each daughter cell must inherit a single copy of each chromosome. The centromere is the locus on each chromosome that ensures the equal distribution of chromosomes during cell division. One essential protein involved in this task is CENP-A^Cse4^, which normally localizes exclusively to centromeres. Here, we investigated where CENP-A^Cse4^ spreads in the genome when parts of its regulatory machinery are removed. We found that CENP-A^Cse4^ becomes mislocalized to promoters, the region upstream of each gene that controls the activity of the gene. Consistent with this, the mislocalization of CENP-A^Cse4^ to promoters leads to problems with gene activity. Our work shows that mislocalization of centromeric proteins can have effects beyond chromosome segregation defects, such as interfering with gene expression on chromosome arms.

## Introduction

The eukaryotic genome is packaged into chromatin, which consists of 147 bp repeating units of DNA wrapped around histone proteins to form nucleosomes [1]. Chromatin is important not only for packaging and protecting DNA, but also for regulating access of genes and other DNA elements to nuclear proteins involved in processes such as transcription, replication, and chromosome segregation. Most nucleosomes are composed of the canonical histone proteins, H2A, H2B, H3, and H4 [2]. However, the behavior and functions of nucleosomes can be altered both by chemically modifying canonical histones through post-translational modifications and by exchanging canonical histones for histone variants that alter nucleosome composition [2]. For example, H2A.Z is a variant of histone H2A and is found at promoter nucleosomes genome-wide where it regulates transcription [2–4]. In contrast, the conserved CENP-A variant (also called CenH3) replaces H3 in nucleosomes exclusively at the centromere where it regulates chromosome segregation [5–7]. Because changes in nucleosome composition can have a major impact on the underlying functions of the genome, it is critical to understand the mechanisms that control the localization of histone modifications and variants.

The genomic incorporation of the budding yeast H2A.Z^Htz1^ (SGD ID: S000005372) histone variant is regulated by the SWR1 (SWR-C) and INO80 (INO80-C) chromatin remodeling complexes [8]. H2A.Z^Htz1^ localizes to intergenic regions, specifically near transcription start sites (TSS) at the +1 and -1 nucleosomes surrounding nucleosome-depleted regions (NDRs) [3, 4, 9–12]. In budding yeast, H2A.Z^Htz1^ nucleosomes are correlated with high nucleosome turnover [13], which is proposed to assist transcriptional initiation or rapid changes between transcriptional states [14–16]. SWR-C incorporates H2A.Z^Htz1^ into nucleosomes by exchanging H2A/H2B dimers for H2A.Z^Htz1^/H2B dimers [17–19]. In contrast, the mechanism of H2A.Z^Htz1^ removal from nucleosomes by INO80-C is less well understood because it has two reported activities that both lead to H2A.Z^Htz1^ exchange, either by swapping H2A.Z^Htz1^/H2B dimers for H2A/H2B dimers [20] or by promoting turnover of the entire nucleosome [8, 19].

The localization of the CENP-A variant is regulated by the histone chaperone HJURP (Scm3 in budding yeast), which is targeted specifically to centromeres [21–25]. Centromeric sequence and size are highly variable throughout eukaryotes and can be specified by either an underlying sequence or through epigenetic inheritance [26, 27]. Despite the diversity of centromeres, CENP-A is a conserved hallmark of all centromeres. The presence of CENP-A directs the formation of the kinetochore, a large protein complex that mediates attachments between the microtubules of the mitotic spindle and the chromosome during cell division [26, 28, 29]. CENP-A mislocalization to euchromatin through overexpression or tethering can lead to ectopic kinetochore formation and genomic instability [30–32]. However, CENP-A mislocalization has not been reported to disrupt other genomic processes [33, 34].

Multiple mechanisms ensure that CENP-A does not mislocalize to euchromatin. A number of chromatin remodelers and histone chaperones are reported to help maintain centromeric chromatin or prevent CENP-A mislocalization, including Fun30, RSC, INO80-C, CAF-1, HIR, FACT, and RbAp48 and SWI/SNF [35–41]. In addition, ubiquitin-mediated proteolysis prevents ectopic CENP-A localization by controlling total CENP-A protein levels [42–45]. In budding yeast, proteolysis of CENP-A^Cse4^ (SGD ID: S000001532) is mediated by an E3 ubiquitin ligase called Psh1 (SGD ID: S000005415) [46, 47]. When CENP-A^Cse4^ is overexpressed in the absence of Psh1-mediated proteolysis [42, 46–48], cells accumulate high levels of CENP-A^Cse4^ in euchromatin. This also results in lethality, although the underlying cause has not been determined [42, 46, 47].

Similar to CENP-A, H2A.Z also contributes to chromosome segregation. In human cells, H2A.Z is found at pericentromeric regions, where it is incorporated at the inner centromere between the CENP-A nucleosome domains, and helps to establish centromeric heterochromatin [49, 50]. Similarly, H2A.Z^Htz1^ is also a component of pericentromeric chromatin in budding yeast, where it localizes to nucleosomes flanking the CENP-A^Cse4^ nucleosome and is important for chromosome segregation through unknown mechanisms [4, 51, 52]. However, it is unclear whether there is a connection between the localization of the histone variants. In human cells, overexpressed CENP-A was found to mislocalize to regions enriched for H2A.Z, although no physical interaction was detected between these two histone variants [33]. In contrast, studies in *S*. *pombe* have shown that CENP-A^Cnp1^ tends to mislocalize to ectopic regions that are depleted of H2A.Z^Htz1^ [53].

We set out to determine whether there are features of euchromatin that normally prevent budding yeast CENP-A misincorporation as well as to identify the functional consequences of CENP-A mislocalization to euchromatin. The identification of euchromatic sites that strongly misincorporate CENP-A may also shed light on the underlying cause of the lethality. To address these questions, we performed the first genome-wide analysis of CENP-A overexpression in the absence of ubiquitin-mediated degradation. We found that overexpressed CENP-A^Cse4^ mislocalizes to promoters that are enriched for NDRs flanked by H2A.Z^Htz1^, and this mislocalization is dramatically enhanced in cells that cannot degrade CENP-A^Cse4^. This localization pattern appears to be due in part to co-opting of INO80-C to incorporate excess CENP-A^Cse4^ into promoter nucleosomes that normally contain H2A.Z^Htz1^. Consistent with this, there was a significant correlation between transcripts that were misregulated in cells lacking H2A.Z^Htz1^ and those with high levels of CENP-A^Cse4^ mislocalization. We also found that a subset of promoters that misincorporate CENP-A^Cse4^ have decreased transcription, which may be the underlying cause of lethality. Together, these data suggest that it is essential that cells regulate CENP-A^Cse4^ localization not only to ensure proper chromosome segregation, but also to protect cells from promoter nucleosome disruption and transcriptional misregulation.

## Results

### Excess CENP-A^Cse4^ mislocalizes to intergenic regions of the genome

**Fig 1:**
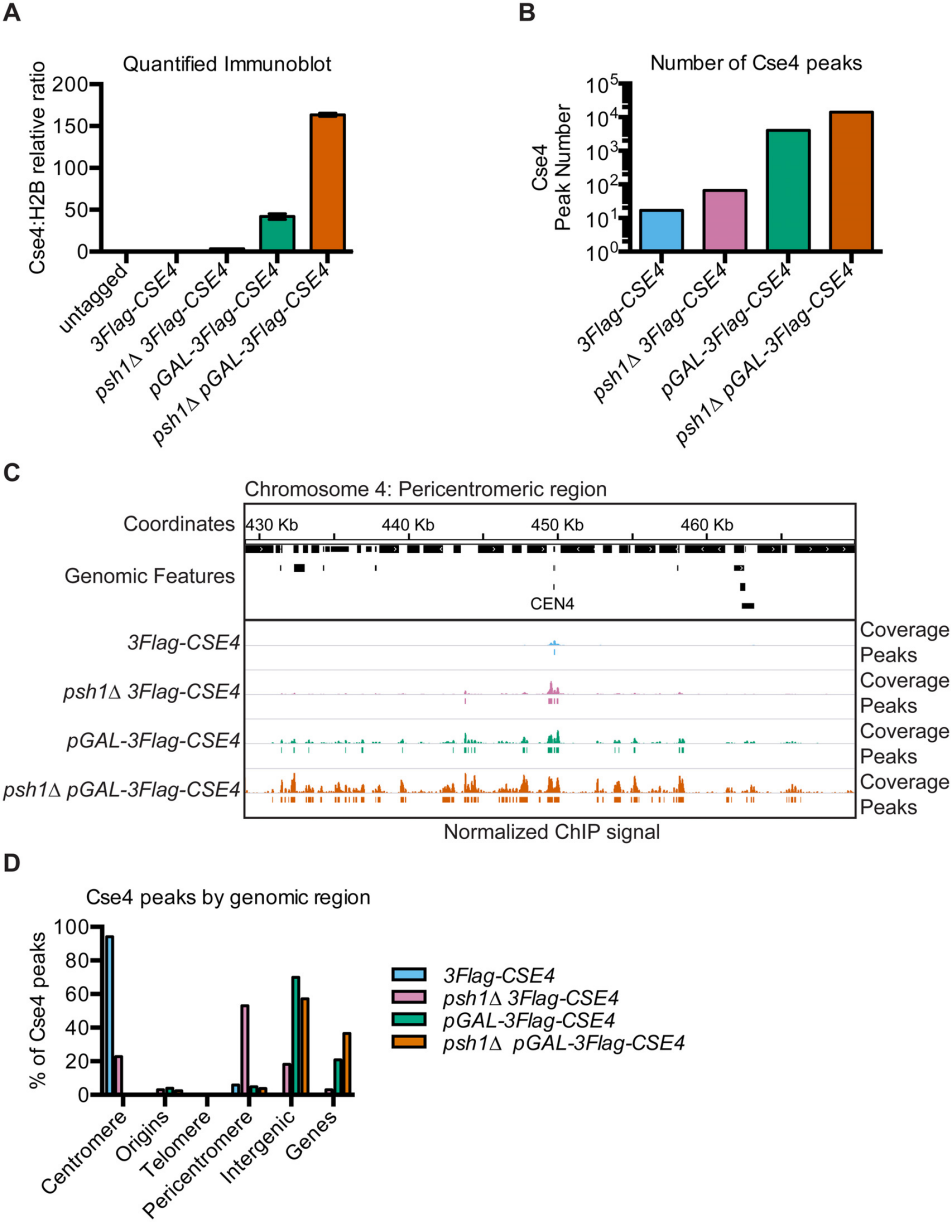
Intergenic regions are the major sites of overexpressed CENP-A^Cse4^ mislocalization. (A) Quantification of CENP-A^Cse4^ levels in MNase-digested chromatin in untagged (SBY3, black), *3Flag-CSE4* (SBY10419, blue), *psh1Δ 3Flag-CSE4* (SBY10484, pink), *pGAL-3Flag-CSE4* (SBY10425, green) and *psh1Δ pGAL-3Flag-Cse4* (SBY10483, orange) strains. The ratio of CENP-A^Cse4^:H2B in the chromatin in each strain was quantified relative to the CENP-A^Cse4^:H2B ratio from the *3Flag-CSE4* (SBY10419) strain. Quantification is based on two biological replicates. Error bars are +/- 1 standard error of the mean (SEM) of the two biological replicates. (B) The total number of CENP-A^Cse4^ peaks called in the indicated strains: *3Flag-CSE4* (SBY10419, blue), *psh1Δ 3Flag-CSE4* (SBY10484, pink), *pGAL-3Flag-CSE4* (SBY10425, green) and *psh1Δ pGAL-3Flag-CSE4* (SBY10483, orange). (C) A representative region of the CENP-A^Cse4^ ChIP-seq coverage on Chromosome 4 between 429,000 base pairs (bp) and 470,000 bp is shown. The CENP-A^Cse4^ ChIP-seq coverage for the strains in (B) is normalized to the coverage at the centromeres after subtracting the input. Peaks are shown as lines below each coverage signal (the cutoff is the average minimum coverage at the centromere in the *3Flag-CSE4* strain). The scale of the normalized coverage is from 0–20,000 for all strains. (D) The percentage of CENP-A^Cse4^ peak centers in each type of genomic region is graphed for each strain, as in (B). The percentage of each feature in the genome is: genes (68.23%), intergenic (27.04%), pericentromeres (2.62%), telomeres (1.16%), origins (0.92%) and centromeres (0.02%).

To identify the precise genomic sites of CENP-A^Cse4^ mislocalization in budding yeast, we performed ChIP-seq on endogenous and overexpressed CENP-A^Cse4^ in the presence and absence of Psh1-mediated proteolysis. All strains contained a fully functional ectopic *3Flag-CSE4* gene integrated at the *URA3* locus under the endogenous promoter and were deleted for the endogenous *CSE4* gene. Cells overexpressing CENP-A^Cse4^ contained an additional copy under the control of the *GAL* promoter (*pGAL-3Flag-CSE4*). As seen previously, CENP-A^Cse4^ overexpression inhibited the growth of WT cells and resulted in lethality in *psh1Δ* cells (S1A Fig) [46, 47]. The growth inhibition correlated with the total amount of chromatin-bound CENP-A^Cse4^ protein (Fig 1A, S1B Fig).

To analyze CENP-A^Cse4^ localization, cells were crosslinked with formaldehyde and the chromatin was isolated and subsequently digested with Micrococcal nuclease (MNase), which cuts linker DNA between nucleosomes. The CENP-A^Cse4^ nucleosomes were purified from the MNase-treated chromatin by immunoprecipitation of 3Flag-Cse4. The amount of CENP-A^Cse4^ recovered in the ChIP samples reflected the starting levels in the chromatin (S1C Fig). The input samples (MNase-digested chromatin) and ChIP samples (3Flag-Cse4-bound chromatin after immunoprecipitation) were made into paired-end sequencing libraries using a modified Solexa library preparation protocol that captures DNA particles down to ~25 bp (S1D Fig) [54, 55]. Paired-end sequencing resulted in greater than 1.5 million reads/sample, with an average read length ranging from 147–164 bp (S1 Table). The mononucleosome-sized sequencing reads from the input and ChIP samples for each strain were mapped to the *S*. *cerevisiae* reference genome version *SacCer3* [56].

The peaks of CENP-A^Cse4^ enrichment genome-wide correlated with the levels of chromatin-bound CENP-A^Cse4^ (Fig 1B, Table 1). Seventeen peaks were identified for the *3Flag-CSE4* strain, representing the sixteen centromeres as well as a peak 150 bp from *CEN9*. A small amount of CENP-A^Cse4^ mislocalization was seen starting in the *psh1Δ* strain with 66 peaks, and was further increased in cells overexpressing CENP-A^Cse4^ with 4043 peaks. The greatest enrichment in the euchromatin was detected in the *psh1Δ* cells overexpressing CENP-A^Cse4^ with 14,199 peaks. An example of the coverage data and corresponding peaks for a representative region around Centromere 4 shows a single centromere peak for the WT strain and additional peaks around the centromere in the other strains (Fig 1C). The increased CENP-A^Cse4^ mislocalization in surrounding euchromatin is especially apparent in the *pGAL-3Flag-CSE4* and *psh1Δ pGAL-3Flag-CSE4* strains that have the highest levels of CENP-A^Cse4^. We independently confirmed the CENP-A^Cse4^ enrichment at *CEN4* and at other representative peaks by ChIP-qPCR (S2A Fig). Our initial analysis also identified a CENP-A^Cse4^ peak at the rDNA locus in all strains. This did not show significant enrichment in the *3Flag-CSE4* strain by ChIP-qPCR but did in the cells with overexpressed CENP-A^Cse4^, similar to previously reported data [46, 57] (S2A Fig). However, due to the difficulty in analyzing this repetitive region by standard mapping algorithms, ChIP coverage of this region was excluded from further computational analyses.

**Table 1:** CENP-A^Cse4^ peak information.

Strain	Peak calling threshold	Average coverage (rDNA removed)	Number of Peaks	Number (and %) of genes with Cse4 peaks in promoter	Number (and %) of genes with Cse4 peaks in 3’ end
*3Flag-CSE4* (SBY10419)	3263	15.3	17	7 (0.106%)	15 (0.228%)
*psh1Δ 3Flag-CSE4* (SBY10484)	3263	122	66	21 (0.319%)	37 (0.563%)
*pGAL-3Flag-CSE4* (SBY10425)	3263	387	4043	3058 (46.5%)	2638 (40.1%)
*psh1Δ pGAL-3Flag-CSE4* (SBY10483)	3263	1696	14199	5921 (90.0%)	5175 (78.7%)

To determine if mislocalized CENP-A^Cse4^ favors certain genomic regions, we analyzed the percentage of CENP-A^Cse4^ peaks in various functional regions of the genome, including centromeres, pericentromeres, telomeres, replication origins, genes, and intergenic regions (Fig 1D). We defined pericentromeres as 20 Kilobases (Kb) flanking each centromere, consistent with the 20–50 Kb size of cohesin enrichment around each centromere in budding yeast [58, 59]. As expected, the majority of CENP-A^Cse4^ peaks in WT cells were at centromeres, with an increase in pericentromeric peaks in the *psh1Δ* mutant. However, the majority of peaks in the strains overexpressing CENP-A^Cse4^ were in the intergenic regions, with a smaller percentage within genes. As intergenic regions make up less than 30% of the entire genome, these data indicate a strong enrichment of CENP-A^Cse4^ in intergenic regions in cells overexpressing CENP-A^Cse4^.

We next asked whether the intergenic enrichment correlates with features known to be associated with centromeres. ChIP-seq of mildly overexpressed CENP-A^Cse4^ previously identified 23 centromere-like regions (CLRs) on chromosome arms that are enriched for mislocalized CENP-A^Cse4^ and other kinetochore proteins [60]. These CLRs share characteristics with centromeric sequences such as having a high AT% and conferring stability to plasmid DNA. As expected, most of the CLRs have CENP-A^Cse4^ peaks in the *psh1Δ pGAL-3Flag-CSE4* strain (S2B Fig). However, CENP-A^Cse4^ was overexpressed to much higher levels in our study (150-fold compared to 3-fold), so the CLRs are a small fraction of the total peaks. Consistent with this, there was also enrichment in low confidence negative control regions (LCNCRs), indicating there is no preference for CLR localization. We also analyzed the AT content of the DNA bound by mislocalized CENP-A^Cse4^, as this is a defining characteristic of centromeric DNA in budding yeast. As expected, CENP-A^Cse4^ peaks were highly enriched for AT nucleotides in the WT strain. However there was only a moderate increase in AT% in the *psh1Δ* strain compared to the input nucleosomes, and almost no AT bias in the strains with overexpressed CENP-A^Cse4^ (S2C–S2F Fig). Together, these data indicate that the mislocalization of CENP-A^Cse4^ is due to a more widespread effect than just centromere-like characteristics.

### Mislocalized CENP-A^Cse4^ is enriched in promoters but is not correlated with basal transcription levels

**Fig 2:**
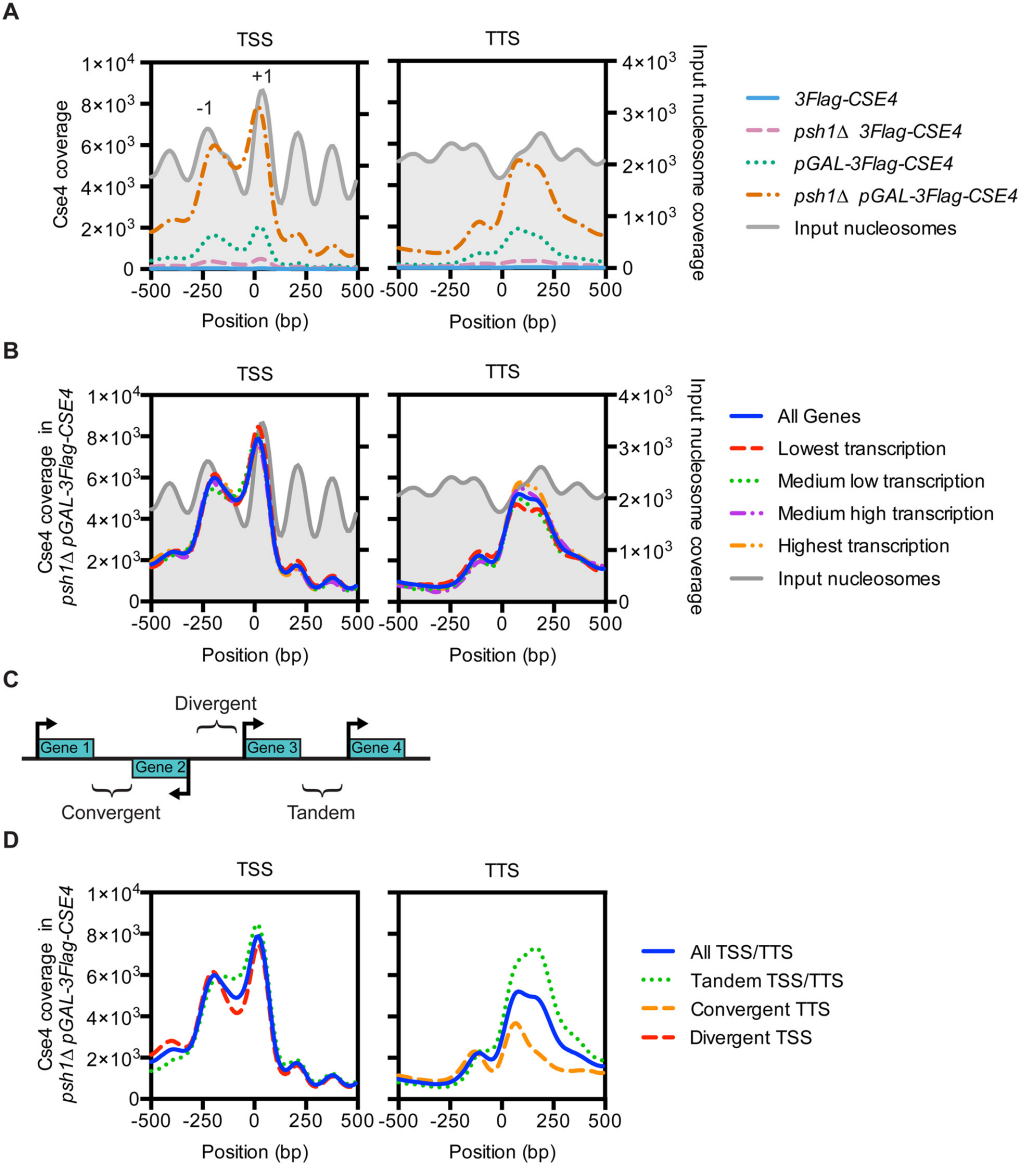
Overexpressed CENP-A^Cse4^ mislocalizes to promoters. (A) Mean CENP-A^Cse4^ ChIP coverage 500 bp upstream and downstream of all transcription start sites (TSS) or transcription termination sites (TTS), for *3Flag-CSE4* (SBY10419, blue), *psh1Δ 3Flag-CSE4* (SBY10484, pink), *pGAL-3Flag-Cse4* (SBY10425, green) and *psh1Δ pGAL-3Flag-Cse4* (SBY10483, orange). The input nucleosome positions are from the input MNase-seq data from the *3Flag-CSE4* strain (SBY10419) and are plotted in grey. (B) Mean CENP-A^Cse4^ ChIP coverage for the *psh1Δ pGAL-3Flag-CSE4* (SBY10483) strain separated by transcription levels of the corresponding genes [62]. (C) Diagram of the classification of the different types of intergenic regions based on the direction of transcription of the flanking genes. (D) Mean CENP-A^Cse4^ ChIP coverage for the *psh1Δ pGAL-3Flag-CSE4* strain (SBY10483) separated by the direction of transcription for the corresponding TSS or TTS.

We next asked whether the intergenic enrichment of overexpressed CENP-A^Cse4^ was specific to either promoters (defined as 500 bp upstream of the transcription start site (TSS)) or transcription terminators (defined as 500 bp downstream of the transcription termination site (TTS)) by calculating the number of peaks in these regions (Table 1). CENP-A^Cse4^ was enriched in both regions when overexpressed, so we more precisely analyzed the pattern by plotting the average coverage in 10 bp windows for regions 500 bp upstream and downstream of all TSS or TTS (the TSS or TTS is plotted at position 0 based on previously reported RNA-seq transcription start positions [61]). In the *psh1Δ* cells overexpressing CENP-A^Cse4^, there was enrichment -200 bp from the TSS and directly over the TSS, which correspond to the -1 and +1 nucleosomes respectively (Fig 2A). At the TTS, CENP-A^Cse4^ was enriched in the nucleosome just after the termination site, and was shifted slightly into the NDR compared to the WT nucleosomes. Although the level of CENP-A^Cse4^ enrichment in the other three strains was much lower overall, the trend is similar in the cells with increased CENP-A^Cse4^. This pattern is reminiscent of the pattern of CENP-A^Cse4^ mislocalization upon deletion of *CAC1* and *HIR1*, which leads to ectopic CENP-A^Cse4^ enrichment at promoters in the presence of Psh1 [38].

We next asked whether the accumulation of CENP-A^Cse4^ in promoters and terminators is associated with the basal level of transcription in WT cells. We plotted CENP-A^Cse4^ enrichment at the TSS and TTS of genes binned into quartiles by the published transcription levels in a WT strain, ranked from lowest transcription to highest transcription [62]. However, there was no correlation between CENP-A^Cse4^ enrichment and the different transcription levels (Fig 2B, S3 Fig). Therefore, the CENP-A^Cse4^ localization to promoters in the *psh1Δ pGAL-3Flag-CSE4* strain was not an artifact of increased chromatin accessibility in areas of high transcription, such as was found in the previously reported CENP-A^Cse4^ ChIP-seq for slightly overexpressed or hypomorphic CENP-A^Cse4^ [63]. We also analyzed whether CENP-A^Cse4^ mislocalization correlated with the direction of transcription of the surrounding genes, since this has been shown for cohesin localization, which is specifically enriched in convergent intergenic regions outside of the pericentromere [58, 64]. We classified the intergenic regions as tandem (between two genes transcribed in the same direction), convergent (between two genes transcribed towards each other), or divergent (between two genes transcribed away from each other) (Fig 2C). In promoters, CENP-A^Cse4^ was enriched at the tandem and divergent genes (Fig 2D, S4 Fig). At the terminators, CENP-A^Cse4^ was enriched at the tandem TTS and depleted at the convergent TTS. Because convergent regions lack promoters, these data are consistent with the enrichment of CENP-A^Cse4^ to promoter regions.

### Mislocalized CENP-A^Cse4^ is found at H2A.Z^Htz1^-enriched nucleosomes flanking NDRs

**Fig 3:**
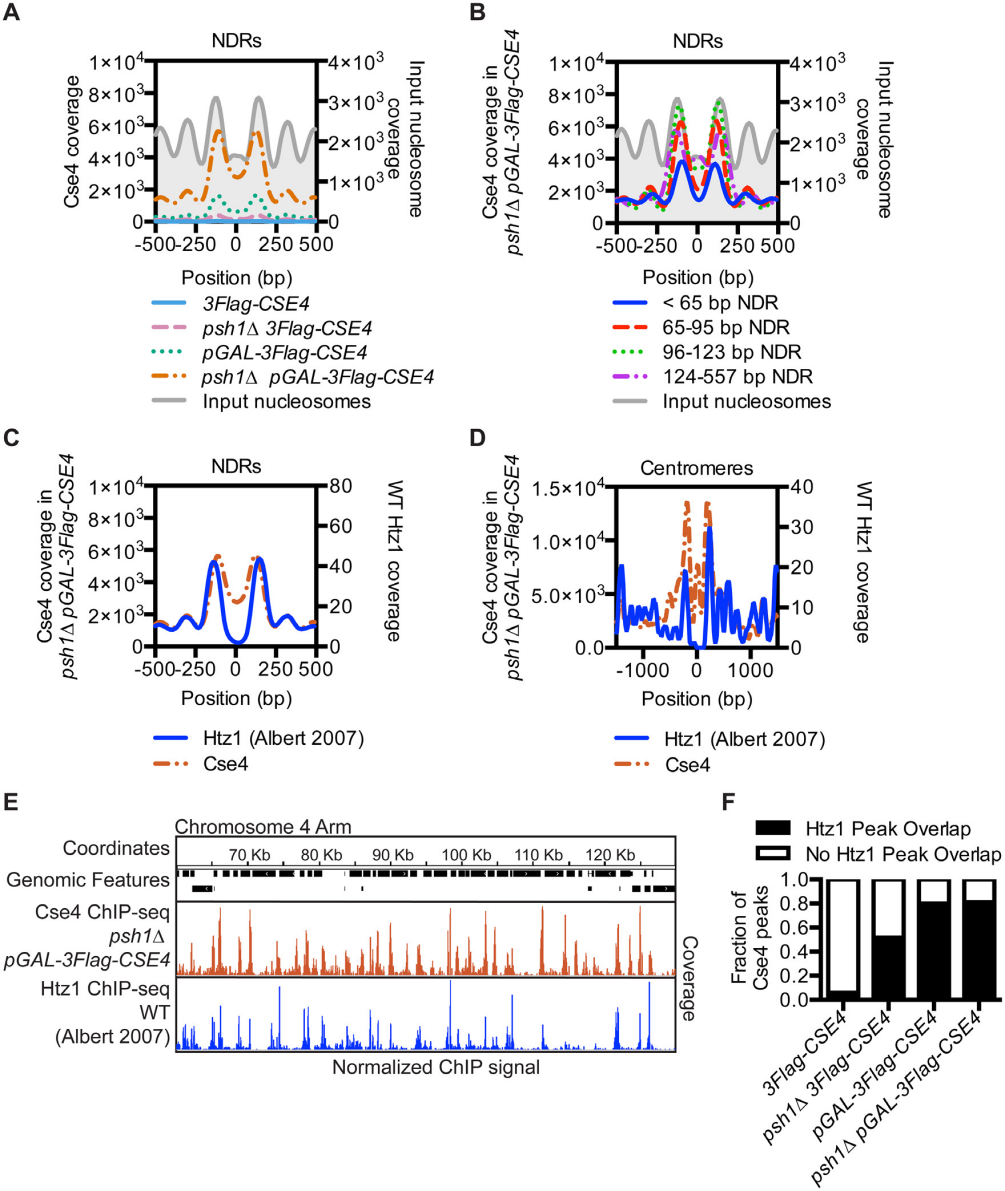
CENP-A^Cse4^ mislocalizes to regions that are enriched for the histone variant H2A.Z^Htz1^. (A) Mean CENP-A^Cse4^ ChIP coverage for the *3Flag-CSE4* (SBY10419, blue), *psh1Δ 3Flag-CSE4* (SBY10484, pink), *pGAL-3Flag-CSE4* (SBY10425, green) and *psh1Δ pGAL-3Flag-CSE4* (SBY10483, orange) strains centered on annotated NDRs [65]. (B) Mean CENP-A^Cse4^ ChIP coverage for the *psh1Δ pGAL-3Flag-CSE4* (SBY10483) strain centered on annotated NDRs, binned by NDR length [65]. (C) Mean CENP-A^Cse4^ ChIP coverage for the *psh1Δ pGAL-3Flag-CSE4* (SBY10483, orange) strain (left y axis) vs. mean H2A.Z^Htz1^ ChIP coverage [4] (blue) (right y axis) at all NDRs. (D) Mean CENP-A^Cse4^ ChIP coverage for the *psh1Δ pGAL-3Flag-CSE4* (SBY10483, orange) strain (left y axis) vs. mean normalized H2A.Z^Htz1^ ChIP coverage [4] (blue) (right y axis) at all centromeres. (E) CENP-A^Cse4^ ChIP coverage for the *psh1Δ pGAL-3Flag-CSE4* (SBY10483, orange) strain and WT H2A.Z^Htz1^ coverage [4] (blue) on the chromosome 4 arm between 60,000 bp and 130,000 bp. The scale of the normalized coverage is from 0–20,000 for the CENP-A^Cse4^ ChIP-seq and from 0–301 for the H2A.Z^Htz1^ ChIP-seq. (F) Overlap between CENP-A^Cse4^ peaks in *3Flag-CSE4* (SBY10419), *psh1Δ 3Flag-CSE4* (SBY10484), *pGAL-3Flag-CSE4* (SBY10425) or *psh1Δ pGAL-3Flag-CSE4* (SBY10483) compared to WT H2A.Z^Htz1^ nucleosomes [4].

Since CENP-A^Cse4^ mislocalization to promoters was not correlated with transcription levels, we looked for another chromatin feature specific to promoters that might enhance CENP-A^Cse4^ incorporation. One characteristic of promoters that is less commonly found at the 3’ ends of genes is the NDR between the -1 and +1 nucleosomes at the TSS [65]. We therefore compared CENP-A^Cse4^ profiles centered on all NDRs and found a strong CENP-A^Cse4^ enrichment in the nucleosomes flanking the NDRs in the *psh1Δ pGAL-3Flag-CSE4* strain (Fig 3A). Because NDRs vary in length up to 557 bp, we asked whether there was a specific NDR length that correlated with CENP-A^Cse4^ mislocalization and found the highest enrichment in NDRs longer than 65 bp (Fig 3B). We obtained similar results when the analysis was centered on the TSS instead of the NDR (S5A–S5D Fig), consistent with the enrichment of CENP-A^Cse4^ in NDR containing promoters.

The localization of CENP-A^Cse4^ to the nucleosomes flanking the NDRs is similar to H2A.Z^Htz1^, the only other histone variant in budding yeast [4]. In addition, the SWR-C chromatin-remodeling complex that incorporates H2A.Z^Htz1^ preferentially binds to NDRs greater than 50 bp [66], similar to the length of NDRs that have the highest CENP-A^Cse4^ enrichment (greater than 65 bp) (Fig 3B). We therefore investigated the relationship between previously reported H2A.Z^Htz1^ localization [4] and the mislocalization of overexpressed CENP-A^Cse4^ in *psh1Δ* cells. There was a striking similarity in their enrichment at NDRs (Fig 3C), as well as a similar trend of co-enrichment in the nucleosomes flanking replication origins (S5E and S5F Fig) and centromeres (Fig 3D, S5G Fig). The CENP-A^Cse4^ coverage at the TSS was also similar to H2A.Z^Htz1^ coverage, while at the TTS CENP-A^Cse4^ was shifted more into the 3’ NDR than H2A.Z^Htz1^ (S6A and S6B Fig). The histone variants exhibited a genome-wide trend to co-localize, as seen in a representative region of the arm of Chromosome 4 (Fig 3E and S6C Fig). There was a high coincidence of overlap between CENP-A^Cse4^ peaks in the experimental strains with H2A.Z^Htz1^ peaks in WT cells, although they were not specifically enriched in any of the genomic features correlated with CENP-A^Cse4^ mislocalization (Fig 3F, S6D and S6E Fig) Together, these data indicate a significant enrichment of misincorporated CENP-A^Cse4^ at sites where H2A.Z^Htz1^ nucleosomes are normally located genome-wide in *psh1Δ* cells overexpressing CENP-A^Cse4^.

### CENP-A^Cse4^ accumulation in chromatin does not depend on H2A.Z^Htz1^

**Fig 4:**
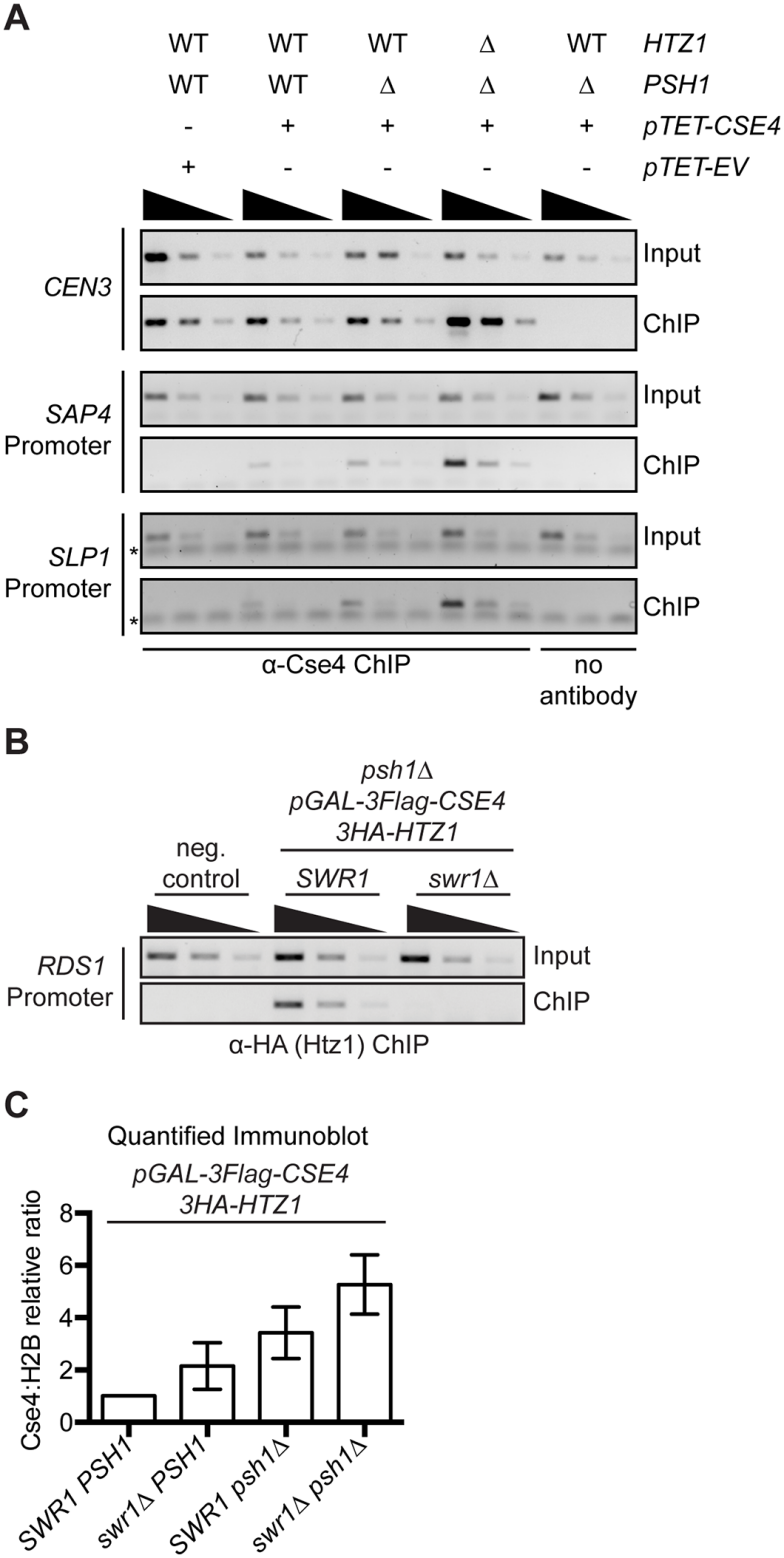
CENP-A^Cse4^ mislocalization does not depend on H2A.Z^Htz1^ incorporation. (A) ChIP was performed with anti-Cse4 antibody on negative control cells (SBY15924), as well as *pTET-CSE4* (SBY15903), *psh1Δ pTET-CSE4* (SBY15904) and *psh1Δ htz1Δ pTET-CSE4* (SBY15906) cells overexpressing CENP-A^Cse4^ for six hours. As a control, we also performed a ChIP experiment with no antibody in *psh1Δ pTET-CSE4* cells. Input dilutions are 1:100, 1:300, 1:100 and ChIP dilutions are 1x, 1:3, 1:9. (B) ChIP-PCR of 3HA-Htz1 at the *RDS1* promoter. Strains used: negative (neg.) control (SBY3), *psh1Δ pGAL-3Flag-CSE4 3HA-HTZ1* (SBY12833), *psh1Δ pGAL-3Flag-CSE4*, *3HA-HTZ1 swr1Δ* (SBY12924). Input dilutions: 1:100, 1:300, 1:900. ChIP dilutions: 1:3, 1:9, 1:21. (C) Relative CENP-A^Cse4^ levels were measured by quantifying the mean chromatin CENP-A^Cse4^:H2B fold change vs. *pGAL-3Flag-CSE4 HA-HTZ1* +/- 1 SEM using quantitative immunoblots of chromatin fraction (n = 3), p = 0.0204 (paired t-test comparing the Cse4:H2B relative ratio in *psh1Δ pGAL-3Flag-CSE4* to *swr1Δ psh1Δ pGAL-3Flag-CSE4*). Strains used were *pGAL-3Flag-CSE4 3HA-HTZ1* (SBY12832), *swr1Δ pGAL-3Flag-CSE4 3HA-HTZ1* (SBY12956), *psh1Δ pGAL-3Flag-CSE4 3HA-HTZ1* (SBY12833) and *swr1Δ psh1Δ pGAL-3Flag-CSE4 3HA-HTZ1* (SBY12924).

The co-localization of the histone variants led us to further analyze their relationship. First, we tested whether H2A.Z^Htz1^ promotes CENP-A^Cse4^ localization by performing ChIP on WT, *psh1Δ*, and *psh1Δ htz1Δ* cells overexpressing CENP-A^Cse4^. *htz1Δ* cells are defective in induction from the *GAL* promoter [67], so we used a tetracycline promoter to control *CSE4* levels. Overexpressed CENP-A^Cse4^ bound to promoter regions in the *psh1Δ htz1Δ* double mutant, at levels similar to or even higher than the *psh1Δ* strain (Fig 4A). These data indicate that H2A.Z^Htz1^ is not required for CENP-A^Cse4^ mislocalization, so we next asked whether the H2A.Z^Htz1^ incorporation machinery is involved. Swr1 (SGD ID: S000002742) is the Swi/Snf related ATPase in SWR-C that deposits H2A.Z^Htz1^ into nucleosomes [11, 19, 68], so we measured the levels of chromatin-bound CENP-A^Cse4^ in *swr1Δ* cells. We confirmed that H2A.Z^Htz1^ was reduced at a previously reported promoter nucleosome locus by ChIP-PCR (Fig 4B)[17, 68, 69]. Similar to our findings with the *htz1Δ* mutant, bulk H2A.Z^Htz1^ was not depleted in the chromatin fraction in *swr1Δ*, but CENP-A^Cse4^ chromatin levels were somewhat higher in the *swr1Δ psh1Δ* cells compared to *psh1Δ* (Fig 4C, S7A and S7B Fig). In addition, there was no change in CENP-A^Cse4^ stability in *swr1Δ* cells (S7C Fig). We also tested whether CENP-A^Cse4^ overexpression in the *psh1Δ* mutant affects H2A.Z^Htz1^ promoter occupancy, but did not detect an effect at the loci analyzed (S7D Fig). However, given that H2A.Z^Htz1^ is estimated to occupy only a small proportion of nucleosomes at any given locus in the population, it may be difficult to detect a significant difference [11, 19]. Together, our data suggest that although ectopic CENP-A^Cse4^ and WT H2A.Z^Htz1^ localize to similar sites, the H2A.Z^Htz1^ incorporation machinery does not promote CENP-A^Cse4^ mislocalization and may instead help to prevent CENP-A^Cse4^ promoter incorporation.

### INO80-C contributes to CENP-A^Cse4^ mislocalization in *psh1Δ* cells

**Fig 5:**
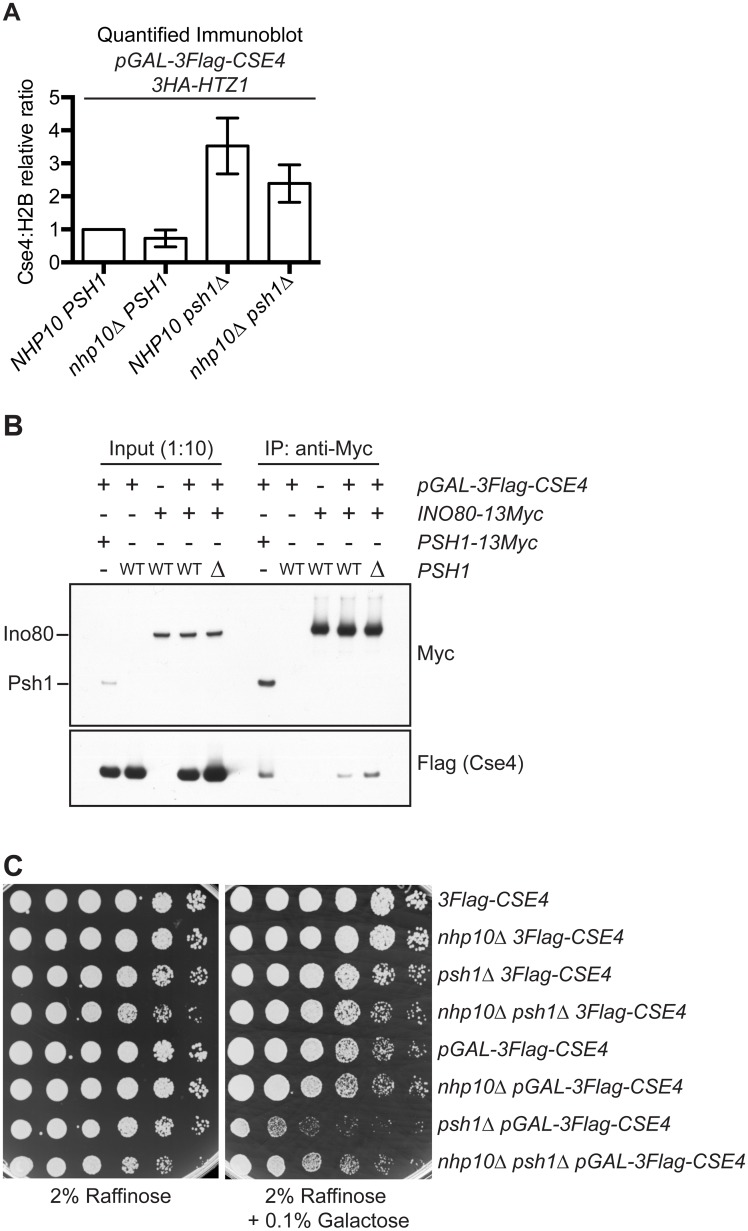
INO80-C contributes to CENP-A^Cse4^ misincorporation. (A) Mean chromatin CENP-A^Cse4^:H2B fold change vs. *pGAL-3Flag-CSE4 HA-HTZ1* strain +/- 1 SEM from quantitative immunoblots of the chromatin fraction. Strains used: *pGAL-3Flag-CSE4 HA-HTZ1* (SBY12832), *nhp10Δ pGAL-3Flag-CSE4 3HA-HTZ1* (SBY12930), *psh1Δ pGAL-3Flag-CSE4 3HA-HTZ1* (SBY12833), and *nhp10Δ psh1Δ pGAL-3Flag-CSE4 3HA-HTZ1* (SBY12959). (n = 3). p = 0.0593 (paired t-test comparing the Cse4:H2B relative ratio in *psh1Δ pGAL-3Flag-CSE4* to *nhp10Δ psh1Δ pGAL-3Flag-CSE4*). (B) Co-Immunoprecipitation (co-IP) of overexpressed 3Flag-Cse4 with either Psh1-13Myc or Ino80-13Myc from the following strains: *PSH1-13Myc pGAL-3Flag-CSE4* (SBY14482), *pGAL-3Flag-CSE4* (SBY9540), *INO80-13Myc* (SBY14527), *INO80-13Myc pGAL-3Flag-CSE4* (SBY14526), *INO80-13Myc psh1Δ pGAL-3Flag-CSE4* (SBY14515). (C) Five-fold serial dilutions of strains *3Flag-CSE4* (SBY10419), *nhp10Δ 3Flag-CSE4* (SBY12958), *psh1Δ 3Flag-CSE4* (SBY10484), *nhp10Δ psh1Δ 3Flag-CSE4* (SBY12928), *pGAL-3Flag-CSE4* (SBY10425), *nhp10Δ pGAL-3Flag-CSE4* (SBY12930), *psh1Δ pGAL-3Flag-CSE4* (SBY10484), and *nhp10Δ psh1Δ pGAL-3Flag-CSE4* (SBY12959) were plated on indicated media.

Since the ectopic localization of CENP-A^Cse4^ does not depend on H2A.Z^Htz1^ incorporation, we asked whether chromatin remodelers that remove H2A.Z^Htz1^ are involved. INO80-C has been reported to act preferentially on H2A.Z^Htz1^-containing +1 nucleosomes and to promote full nucleosome turnover [19, 20]. We therefore hypothesized that CENP-A^Cse4^ might be incorporated into chromatin when canonical H3 is removed by INO80-C-mediated nucleosome turnover. Previous work showed that deletion of the ATPase Ino80 (SGD ID: S000003118) leads to a global alteration of H2A.Z^Htz1^ localization patterns genome-wide without affecting the overall levels of H2A.Z^Htz1^ incorporation in the genome [20, 70]. However, this deletion mutant is not viable in the strain background we used in this study [71]. We therefore used a deletion of *NHP10* (SGD ID: S000002160), a non-essential INO80-C subunit that facilitates binding to nucleosomes and DNA, but that does not affect catalytic activity *in vitro* [72–74]. To analyze CENP-A^Cse4^ levels, we performed chromatin fractionation in WT and *nhp10Δ* cells overexpressing CENP-A^Cse4^. Similar to previously reported work, we did not detect a change in total H2A.Z^Htz1^ levels in the chromatin in the *nhp10Δ* strain (S8A and S8B Fig)[20, 70]. However, CENP-A^Cse4^ chromatin levels were somewhat reduced when *NHP10* was deleted (Fig 5A and S8B Fig), suggesting that INO80-C histone exchange activity contributes to CENP-A^Cse4^ misincorporation. To more directly test this possibility, we asked whether Ino80 associates with CENP-A^Cse4^ *in vivo*. CENP-A^Cse4^ co-immunoprecipitated with Ino80 (Fig 5B), and this interaction increased in the absence of Psh1. To determine how this affects cell viability, we also analyzed the growth of *nhp10Δ* mutant cells overexpressing CENP-A^Cse4^. Although strong CENP-A^Cse4^ overexpression is lethal to *psh1Δ* cells regardless of the presence of *NHP10* (S8C Fig), a deletion of *NHP10* improved the growth of *psh1Δ* mutant cells that were moderately overexpressing CENP-A^Cse4^ (Fig 5C). We confirmed these effects were not due to altered levels or stability of CENP-A^Cse4^ in *nhp10Δ* mutant cells (S8D and S8E Fig). Together, these data suggest that at least some of the ectopic CENP-A^Cse4^ deposition is likely coupled to the chromatin remodeling activity of INO80-C.

### Mislocalized CENP-A^Cse4^ perturbs transcription in the absence of Psh1

**Fig 6:**
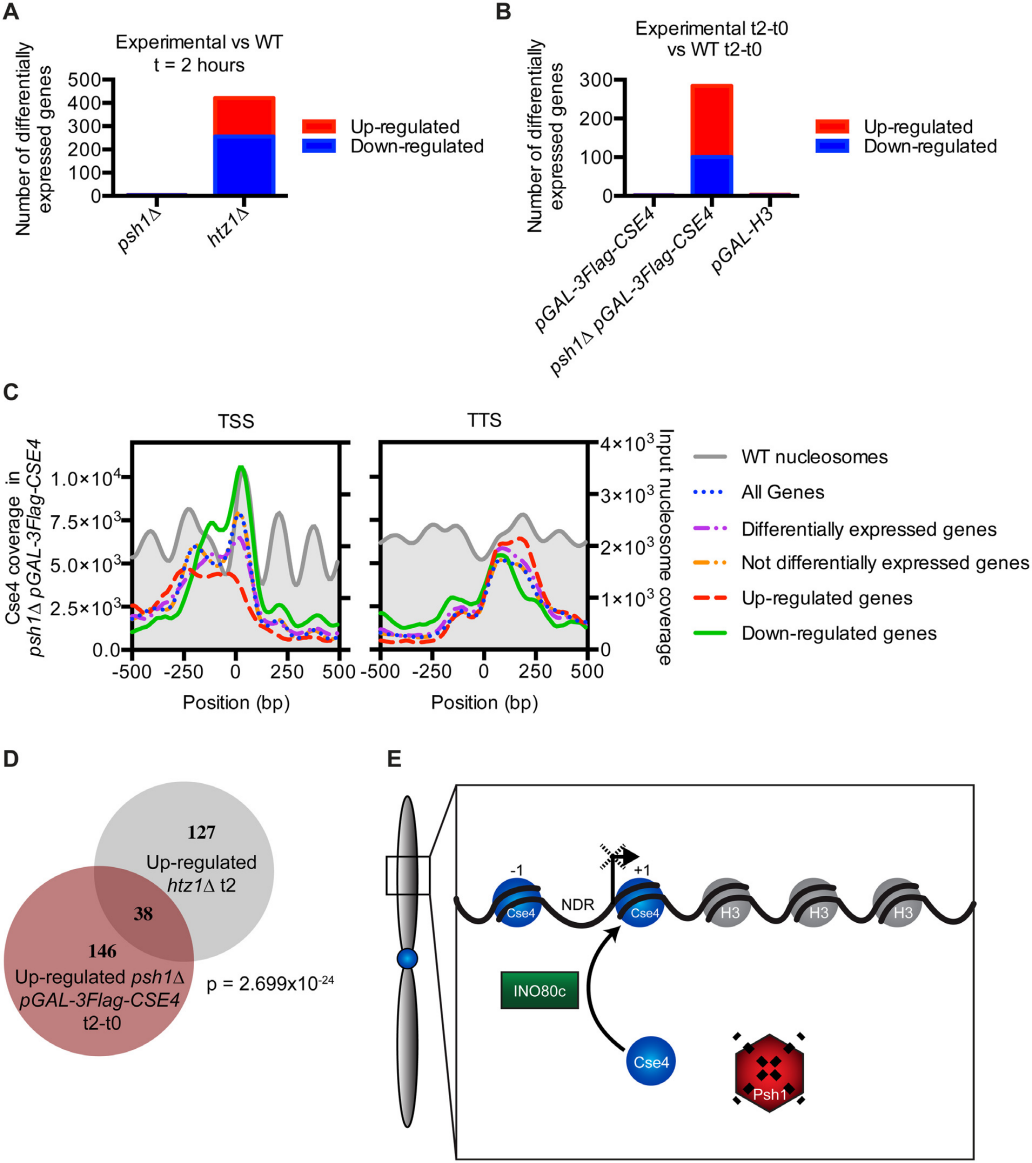
CENP-A^Cse4^ mislocalization to promoters alters transcription. (A) Graph of the number of transcripts significantly increased or decreased compared to the WT strain by RNA-seq of *psh1Δ* and *htz1Δ* cells at t = 2 hours. Differential expression analysis statistics were performed using edgeR [78]. (B) Graph of the number of transcripts significantly increased or decreased at t = 2 hours compared to t = 0 in *pGAL-3Flag-CSE4*, *psh1Δ pGAL-3Flag-CSE4*, and *pGAL-H3* that are not also changed at t = 2 hours compared to t = 0 in the WT strain. (C) TSS and TTS profiles of CENP-A^Cse4^ enrichment based on the measured transcriptional changes by RNA-seq in the *psh1Δ pGAL-3Flag-CSE4* strain. (D) Proportional Venn diagram of genes up-regulated in *psh1Δ pGAL-3Flag-CSE4* at t = 2 hours and genes up-regulated in *htz1Δ* at t = 2 hours. p = 2.699x10^-24^ (p-value from a cumulative hypergeometric distribution test, which represents the probability of the number of genes overlapped or greater between the two strains.) (E) Model: At chromosome arms, INO80-C-mediated full nucleosome turnover may lead to CENP-A^Cse4^ deposition and H2A.Z^Htz1^ removal. Psh1 blocks stable CENP-A^Cse4^ promoter incorporation by ubiquitylating mislocalized CENP-A^Cse4^ and targeting it for degradation. Mislocalization of CENP-A^Cse4^ to promoters leads to misregulation of a subset of downstream genes, which could affect survival of these cells.

The mislocalization of CENP-A^Cse4^ to promoters suggested that it could lead to transcriptional changes in the downstream genes. In addition, the relationship between CENP-A^Cse4^ incorporation and H2A.Z^Htz1^ removal by INO80-C suggested that any transcriptional changes might correlate with those in *htz1Δ* cells. We therefore performed RNA-seq on WT, *psh1Δ*, *pGAL-3Flag-CSE4*, *psh1Δ pGAL-3Flag-CSE4* and *htz1Δ* strains that were treated with galactose for two hours. As a control, we also included a *pGAL-H3* strain to ensure any effects were specific to CENP-A^Cse4^ overexpression and not just an effect of increased histone turnover. Cells containing just a *PSH1* deletion or overexpressing CENP-A^Cse4^ or H3 had very little change in transcription (Fig 6A and 6B). However, a large number of genes were misregulated in *psh1Δ* cells overexpressing CENP-A^Cse4^, as well as in *htz1Δ* cells as previously described [75, 76]. We confirmed that these gene expression changes were not due to an indirect effect of CENP-A^Cse4^ mislocalization to the rDNA by measuring the rDNA copy number and rRNA transcript levels, which were not significantly different between the strains (S9A and S9B Fig). We also confirmed that the differentially transcribed genes in the *psh1Δ pGAL-3Flag-CSE4* strain are not a consequence of altered cell cycle progression [47, 77] (S9C Fig).

To determine whether CENP-A^Cse4^ mislocalization to promoters correlates with transcriptional misregulation of downstream genes, we compared the promoters with CENP-A^Cse4^ peaks to the genes showing altered transcription in the *psh1Δ* strain overexpressing CENP-A^Cse4^. While there was a significant overlap (p = 0.0009, hypergeometric distribution) between the down-regulated genes and those with promoter CENP-A^Cse4^ peaks (S9D Fig), the vast majority of genes with CENP-A^Cse4^ promoter peaks do not have changes in transcription. This is similar to the relationship between H2A.Z^Htz1^ peaks and the genes that are differentially regulated in *htz1Δ* [9], confirming that changes in the histone composition of promoters does not always lead to direct transcriptional effects. However, the downregulated genes have much higher CENP-A^Cse4^ coverage at the +1 nucleosome compared to other promoters, suggesting that both the amount and position of CENP-A^Cse4^ misincorporation may determine which downstream genes become misregulated (Fig 6C). Analysis of transcription factor binding sites enriched at promoters of the downregulated genes with CENP-A^Cse4^ promoter peaks identified Cse2 (SGDID: S000005293) as the most significantly enriched transcription factor (S2 File). Cse2 is a subunit of the RNA Polymerase II Mediator complex, and has also been shown to be required for chromosome segregation [79, 80], leading to the possibility that the transcriptional defects are correlated with altered Cse2 function.

Given the relationship between CENP-A^Cse4^ and H2A.Z^Htz1^ localization, we also asked whether there was a correlation between the transcriptional changes in *psh1Δ pGAL-3Flag-CSE4* and *htz1Δ* mutant cells. Interestingly, there was a significant overlap between the genes that increased transcription in both strains (Fig 6D), and these were also enriched for CENP-A^Cse4^ in the NDR (S9E Fig). We analyzed the promoters of the affected genes for common transcription factors and found 24 that are enriched at the promoters of these genes (S2 File), so the underlying mechanism for the misregulation is not clearly associated with one factor. However, these data are consistent with the relationship between CENP-A^Cse4^ mislocalization and the INO80-C chromatin remodeling machinery that controls H2A.Z^Htz1^.

## Discussion

In this study, we performed the first genome-wide localization of the centromeric histone variant CENP-A^Cse4^ in the absence of Psh1-mediated proteolysis and found that it mislocalizes to intergenic regions when overexpressed. There was a significant correlation between the sites of CENP-A^Cse4^ mislocalization and nucleosomes that normally incorporate the H2A.Z^Htz1^ variant. Consistent with this, we found that INO80-C, which acts on H2A.Z^Htz1^ nucleosomes, also contributes to the ectopic localization of CENP-A^Cse4^, identifying another mechanism that promotes CENP-A^Cse4^ mislocalization. We also found that the number of CENP-A^Cse4^ ectopic peaks is significantly enhanced and leads to transcriptional defects when Psh1 is absent, underscoring the importance of proteolysis in maintaining genome stability through the exclusive localization of the centromeric histone variant.

The intergenic mislocalization of CENP-A^Cse4^ is similar to what has been observed with mild CENP-A^Cse4^ overexpression [54, 60] although we found that the mislocalization of overexpressed CENP-A^Cse4^ is much stronger in the absence of proteolysis. In human cells, CENP-A overexpression misincorporates at CTCF binding sites, which are associated with the histone variants H2A.Z and H3.3 and have high levels of histone turnover [33]. In budding yeast, the connection between histone turnover and CENP-A^Cse4^ mislocalization is less clear. High histone turnover and more open chromatin have been shown to be permissive for CENP-A^Cse4^ mislocalization [54, 60]. This is consistent with our results, as promoters have a higher level of turnover than intragenic regions [13]. However, a *caf1Δ hir1Δ* double mutant that decreases histone turnover genome-wide still mislocalizes even endogenous levels of CENP-A^Cse4^ to promoters [38]. Therefore, histone turnover is not strictly required for CENP-A^Cse4^ mislocalization and there must be additional mechanisms that promote the ectopic deposition of CENP-A^Cse4^.

### INO80-C promotes CENP-A^Cse4^ mislocalization

We identified a strong similarity between H2A.Z^Htz1^ localization and CENP-A^Cse4^ mislocalization in nucleosomes flanking NDRs, such as replication origins, centromeres, and +1 nucleosomes at promoters. We also found that INO80-C contributes to CENP-A^Cse4^ mislocalization. CENP-A^Cse4^ co-immunoprecipitates with INO80-C, and this interaction is increased in the *psh1Δ* mutant where there are higher levels of CENP-A^Cse4^. Consistent with this, an *nhp10Δ* mutant reduced the ectopic localization and partially rescued the growth defect of the *psh1Δ* mutant when CENP-A^Cse4^ was overexpressed. However, *nhp10Δ* does not fully rescue the lethality or ectopic deposition, so additional chromatin remodelers or histone chaperones must also contribute to ectopic CENP-A^Cse4^ incorporation. In humans, the chaperone activity of DAXX is involved in CENP-A deposition in euchromatin [33], but there is no ortholog of this protein in budding yeast.

H2A.Z^Htz1^ localization to nucleosomes flanking NDRs requires SWR-C binding, and SWR-C enrichment is increased with longer NDRs *in vivo* [66]. Similarly, we found that CENP-A^Cse4^ is enriched at longer NDRs. However, we determined that H2A.Z^Htz1^ and SWR-C are not required for CENP-A^Cse4^ deposition. Our work is instead consistent with the possibility that the two yeast histone variants could have an antagonistic relationship, such that they are found at the same places in the genome, but never at the same time. This is reminiscent of the relationship between CENP-A^Cnp1^ and H2A.Z^Htz1^ in fission yeast, where CENP-A^Cnp1^ forms neocentromeres in regions with low H2A.Z^Htz1^ when the endogenous centromere is deleted [53]. However, we detect CENP-A^Cse4^ mislocalization at nucleosomes that normally have high H2A.Z^Htz1^ enrichment. We speculate that this is due to different mechanisms leading to ectopic deposition. In fission yeast, the ectopic CENP-A^Cnp1^ localization to neocentromeres depended on the centromeric chaperone [53], while our data suggests a role for INO80-C in the ectopic deposition of highly expressed CENP-A^Cse4^. Given that INO80-C acts in opposition to SWR-C to remove H2A.Z^Htz1^ from nucleosomes, we propose that the full nucleosome turnover activity of INO80-C leads to the removal of H3 and the incorporation of CENP-A^Cse4^ into promoter nucleosomes (Fig 6E). This model explains both the co-localization of the histone variants and the potentially antagonistic relationship between H2A.Z^Htz1^ and CENP-A^Cse4^ in the chromatin.

### Psh1 acts on CENP-A^Cse4^ throughout the euchromatin

Although there is a significantly higher level of euchromatic CENP-A^Cse4^ in the absence of Psh1, the locations of the ectopic nucleosome positions are similar regardless of Psh1 activity. In both cases, overexpressed CENP-A^Cse4^ is enriched intergenically, suggesting that Psh1 does not have preferential sites of action genome-wide. However, CENP-A^Cse4^ was not significantly incorporated into genes even in the absence of Psh1, suggesting that additional mechanisms control its localization. We previously showed that the FACT complex, which was recently demonstrated to remove H2A.Z^Htz1^ from genes, interacts with Psh1 to facilitate CENP-A^Cse4^ degradation [47, 70, 81]. However, FACT does not interact with CENP-A^Cse4^ in the absence of Psh1 [47]. One possibility is that FACT could indirectly antagonize CENP-A^Cse4^ mislocalization into genes by ensuring that H3 is quickly reincorporated into nucleosomes following transcription, similar to its role in fission yeast [40]. In the future, it will be important to understand how intragenic regions are protected from CENP-A^Cse4^ deposition.

### CENP-A^Cse4^ mislocalization causes defects in transcription

For the first time in any organism, we detected large-scale changes in transcription when CENP-A mislocalized to euchromatin. This only occurred in cells lacking Psh1, and the downregulated genes had very high levels of CENP-A^Cse4^ in their promoters. This suggests that strong misincorporation of CENP-A^Cse4^ at a promoter may be required to cause transcriptional defects, and may explain why this has not been previously observed. The levels of CENP-A^Cse4^ overexpression achieved in the absence of proteolysis are much higher than previous studies that have analyzed CENP-A^Cse4^ mislocalization. It is not clear whether mislocalization of CENP-A^Cse4^ at a given promoter is sufficient to directly decrease transcription. We found a significant enrichment of the Cse2 transcription factor in the promoters of the downregulated genes, leading to the intriguing possibility that CENP-A^Cse4^ incorporation alters Cse2 function at a subset of genes to inhibit transcription. It is interesting to note that Cse2 and Cse4 were identified in the same genetic screen for mutants in chromosome segregation [5, 79], and it will be important to further explore their relationship in the future.

We also identified genes that increased transcription when CENP-A^Cse4^ was mislocalized, and these significantly overlap with those altered in *htz1Δ* mutant cells. This further confirms the potential antagonistic relationship between the yeast histone variants, and suggests that high levels of CENP-A^Cse4^ may lead to similar chromatin changes at a subset of promoters as cells lacking H2A.Z^Htz1^. The underlying mechanism for why only a fraction of promoters that contain H2A.Z^Htz1^ are transcriptionally up-regulated in its absence is not known. We speculate that a change in nucleosome positioning or stability occurs at these promoters that facilitates the access of transcriptional machinery. Consistent with this, we found that the up-regulated gene promoters have CENP-A^Cse4^ enrichment within rather than flanking the NDR and lack strong +1 enrichment.

We found that regulating the levels and localization of the centromeric histone variant is critical to prevent transcriptional misregulation in budding yeast. Although CENP-A mislocalization leads to the formation of ectopic kinetochores in other organisms, we have not been able to determine whether this occurs in budding yeast due to the difficulty of detecting ectopic kinetochores [47]. Our work suggests the possibility that transcriptional defects due to the mislocalization of CENP-A^Cse4^ in the absence of proteolysis may be the underlying cause of lethality in these cells. These data highlight the need to accurately regulate the localization of the centromeric histone variant CENP-A^Cse4^ to both ensure genomic stability through its centromeric functions, as well as to prevent the disruption of euchromatic functions.

## Materials and Methods

### Yeast strain construction and microbial techniques

Microbial techniques and media were as described [82, 83]. For all experiments involving induction of *pGAL-3Flag-CSE4* or *pGAL-H3*, budding yeast cells of indicated strains were grown to log phase (OD 0.55–0.8, Bio-Rad SmartSpec 3000) in lactic acid media at 23°C and induced for 2 hours with 2% galactose. Yeast strains were constructed using standard genetic techniques. Epitope-tagged proteins were constructed using either a PCR integration technique [84] or by the integration of plasmids after restriction digestion. Specific plasmids and yeast strains used in this study are described in the S2 and S3 Tables.

### General protein techniques

Protein extracts to check total CENP-A^Cse4^ levels were prepared as described [85]. Immunoblots using chemiluminescence were performed as previously described [85]. For all immunoblots, the antibody dilutions were as follows: Mouse anti-Pgk1 monoclonal antibodies (Invitrogen Catalog # 459250) at a 1:10,000 dilution were used as a loading control. Mouse anti-Flag M2 monoclonal antibodies (Sigma-Aldrich Catalog # F3165) were used at a 1:3000 dilution, Mouse anti-HA 12CA5 monoclonal antibodies (Roche Catalog # 1-583-816) were used at a 1:10,000 dilution, and rabbit anti-H2B polyclonal antibodies (Active Motif Catalog # 39237) were used at a 1:3,000 dilution. Mouse anti-Myc 9E10 monoclonal antibodies were used at a 1:10,000 dilution (Covance Catalog # MMS-150R). Co-IP experiments were performed as previously described [81] for Psh1-Myc and Ino80-Myc strains using 5ul Protein G Dynabeads conjugated with 1.5ul anti-Myc (A-14, SC-789) and run on a gradient SDS-PAGE gel. Quantitative immunoblots were carried out according to [86] with the modification of using 4% non-fat milk in PBS as the blocking agent for the anti-Flag immunoblot. Briefly, IRDye anti-mouse and anti-rabbit secondary antibodies from LI-COR were used at a 1:15,000 dilution. The immunoblots were imaged on a LI-COR imaging system, and the protein levels were quantified using Image Studio Lite.

### Chromatin fractionation assay

Chromatin fractionation assays were performed as described [81], followed by quantitative immunoblots. The mean and SEM of three independent experiments is reported. anti-PGK1 was used as a marker and loading control for the soluble fraction, and anti-H2B was used as a marker and loading control for the chromatin fraction. The Cse4:H2B and H2A.Z:H2B ratios were normalized to the *pGAL-3Flag-CSE4* strain. Note that the levels of H2A.Z^Htz1^ and H2B are somewhat variable between strains. This may be due to differential susceptibility of the cell wall to zymolyase digestion during the chromatin fractionation procedure, which seems to vary between strains. To control for this, we used H2B to determine the level of total chromatin in each condition.

### ChIP-seq

3Flag-Cse4-containing nucleosomes were isolated by ChIP of 3Flag-Cse4 using monoclonal anti-Flag M2 antibodies (Sigma-Aldrich Catalog # F3165). ChIPs were performed with Micrococcal nuclease (MNase, Worthington Biochemical Corporation Catalog # LS004798)-treated chromatin as described [55] with the following addition. Before nuclei isolation, proteins were crosslinked to DNA with 1% formaldehyde for 15 minutes. Crosslinks were then reversed before DNA extraction by the addition of 1% SDS and an overnight incubation at 65°C [87]. DNA was extracted using phenol:chloroform extraction and ethanol precipitation, and was treated with RNAse and purified using a Qiagen Reaction Clean-up kit before library construction. Paired-end sequencing libraries of both input DNA from MNase-digested chromatin and 3Flag-Cse4 ChIP DNA were prepared using a modified Solexa library preparation protocol that captures DNA particles down to ~25 bp [55]. Cluster generation, followed by 25 cycles of paired-end sequencing on an Illumina HiSeq 2000, was performed by the Fred Hutchinson Cancer Research Center Genomics Shared Resource facility, resulting in 24 bp paired end reads. Base calling was performed using Illumina's Real Time Analysis software v1.13.48.0. Raw FASTQ sequence files were deposited in the NCBI GEO Series GSE69696.

### Identification of CENP-A^Cse4^-enriched loci from ChIP-seq data

Raw reads (passing Solexa quality test) were mapped to the *S*. *cerevisiae* reference genome version SacCer3 (*Saccharomyces* Genome Database (SGD)/UCSC) using the Burrows-Wheeler Aligner (BWA) [88]. The resulting Binary Sequence Alignment/Map (BAM) files were filtered for proper pairs with a mapping score > = 30 using samtools [89]. Mononucleosomes were identified as paired-end reads with insert sizes between 50 bp and 240 bp using R Bioconductor packages GenomicRanges, rtracklayer, Rsamtools, nucleR, and the UCSC SacCer3 reference genome [56, 90–92]. ChIP reads were compared to the input reads for each strain using the Dynamic Analysis of Nucleosome and Protein Occupancy by Sequencing, version 2 (DANPOS2) function Dpos with background subtraction [93], and the background-subtracted ChIP signal was normalized to the coverage at centromeric regions for each strain, which contains a CENP-A^Cse4^ nucleosome throughout the cell cycle [87], and smoothed using the default DANPOS2 Dpos smoothing parameters [93]. The resulting normalized coverage data was visualized using the Integrated Genomics Viewer (IGV) [94, 95]. Wiggle track format (WIG) files of the normalized coverage for each sample in 10 bp steps are available under NCBI GEO Series GSE69696.

To identify genomic loci enriched for CENP-A^Cse4^, we analyzed the coverage relative to the centromere. Although CENP-A^Cse4^ is constitutively localized to the centromere [96], its coverage at the centromere is under-represented relative to other genomic regions. This effect is likely due to the decreased solubility of the centromere to MNase digestion due to kinetochore protein binding, which makes it possible for other genomic regions to appear enriched above its occupancy at the centromere [54]. We called peaks of CENP-A^Cse4^ occupancy in each strain as any region where the CENP-A^Cse4^ enrichment was above the threshold of the minimum average coverage at any centromere in the *3Flag-CSE4* strain using R Bioconductor packages Genomic Ranges, rtracklayer, and the UCSC SacCer3 reference genome [56, 90–92] and the DANPOS2 function Dtriple to call peaks without any further normalization or smoothing [93]. rDNA ChIP coverage was set to 0 before peak calling due to the high copy number of this region, and this locus was excluded from subsequent computational analyses. Input nucleosome peaks were also called using DANPOS2 [93]. Browser Extensible Data (BED) files of the called peaks for each sample are available at NCBI GEO Series GSE69696.

### Overlap of CENP-A^Cse4^ peaks with genomic regions

Genomic regions were annotated using the following strategy: *Saccharomyces* Genome Database (SGD) annotations of the SacCer3 genome were used to call regions of centromeres, pericentromeres, telomeres, origins of replication, genes, and intergenic regions in that order, such that each base was assigned to only the first overlapping region type. To analyze the percentage of peaks from each strain in each genomic region, 1 bp regions at the center of each CENP-A^Cse4^ peak were overlapped with each region so that each peak was counted only once using R Bioconductor packages Genomic Ranges, rtracklayer, and UCSC SacCer3 [56, 90–92]. The same analysis was performed with CENP-A^Cse4^ peaks that either did or did not overlap WT H2A.Z^Htz1^ peaks.

### Meta-analysis of CENP-A^Cse4^ and H2A.Z^Htz1^ enrichment at gene ends and other genomic loci

We analyzed mean CENP-A^Cse4^ and H2A.Z^Htz1^ enrichment at the starts and ends of genes as well as centered on NDRs, origins of replication, or centromeres using the DANPOS2 profile function [65, 93]. H2A.Z^Htz1^ ChIP data is from [4]. H2A.Z^Htz1^ coverage was calculated from the mapped reads with greater than 90% identity using the DANPOS2 function dpos with the default parameters [93], after lifting over the coordinates to the SacCer3 genome using R Bioconductor packages Genomic Ranges, rtracklayer, and UCSC SacCer3 [56, 90–92]. For the analysis of the transcription start sites (TSS) and transcription termination sites (TTS), the mean CENP-A^Cse4^ or H2A.Z^Htz1^ coverage in 10 bp windows was calculated for 500 bp upstream and downstream of 3987 transcripts using custom gene files modified to use experimentally derived TSS data instead of open reading frame (ORF) start sites from Nagalakshmi et al, 2008 (GSE11209) [61]. For the analysis of specific groups of genes, the gene file was divided into the specified bins using R Bioconductor packages before using the DANPOS2 function. For NDRs, origins, and centromeres, DANPOS2 profile was run centered on the genomic features using bed files containing either each NDR [65], origin (from SacCer3 annotation) or centromere (from SacCer3 annotation). All plots were made using GraphPad Prism version 6.0 for OSX, GraphPad Software, La Jolla California USA, www.graphpad.com.

### Comparison of CENP-A^Cse4^ and H2A.Z^Htz1^ nucleosome localization

Coverage data was visualized using IGV [94, 95]. The fraction of overlap between CENP-A^Cse4^ peaks for each strain and reported H2A.Z^Htz1^ peaks (coarse grain nucleosome positions) from wild-type (WT) cells [4] was calculated using R Bioconductor packages GenomicRanges and rtracklayer [90–92].

### ChIP-PCR with sonication for H2A.Z^Htz1^ and *pTet-CSE4*

ChIP was performed from 50 ml formaldehyde-crosslinked cultures as described [97]. Chromatin was fragmented by sonication to approximately 500 bp fragments. For HA-Htz1 ChIP, 3HA-Htz1 was immunoprecipitated using anti-HA (12CA5) antibodies (Roche). 3-fold serial dilutions of the Input (1:100, 1:300, 1:900) and ChIP (1:3, 1:9, 1:27) DNA were used for PCR reactions to detect the amount of DNA pulled down with 3HA-Htz1 in each strain [97] and were analyzed on 1.4% agarose gels. Primers for the *RDS1* promoter are from [69] and are listed in S4 Table. For CENP-A^Cse4^ ChIP, CENP-A^Cse4^ was immunoprecipitated using anti-Cse4 (235N) antibodies [6] from strains with CENP-A^Cse4^ expression induced from an inducible tetracycline repressed promoter [98] after a 6-hour washout of doxycycline (5ug/ml) in YC-URA media. 3-fold serial dilutions of the Input (1:100, 1:300, 1:900) and ChIP (1x, 1:3, 1:9) DNA were used for PCR reactions at *CEN3* [87], the *SAP4* promoter and the *SLP1* promoter.

### RNA-seq

Total RNA was extracted from each sample using a hot acid phenol extraction protocol [99], followed by DNAse I treatment (Invitrogen Amplification Grade) phenol:chloroform extraction, and ethanol precipitation. Two or three independent biological replicates of each genotype were used. Total RNA integrity was checked using an Agilent 2200 TapeStation (Agilent Technologies, Inc., Santa Clara, CA) and quantified using a Trinean DropSense96 spectrophotometer (Caliper Life Sciences, Hopkinton, MA). RNA-seq libraries were prepared from total RNA using the TruSeq RNA Sample Prep v2 Kit (Illumina, Inc., San Diego, CA, USA) and a Sciclone NGSx Workstation (PerkinElmer, Waltham, MA, USA). Library size distributions were validated using an Agilent 2200 TapeStation (Agilent Technologies, Santa Clara, CA, USA). Additional library QC, blending of pooled indexed libraries, and cluster optimization were performed using Life Technologies’ Invitrogen Qubit^®^ 2.0 Fluorometer (Life Technologies-Invitrogen, Carlsbad, CA, USA).

RNA-seq libraries were pooled (18-plex) and clustered onto a flow cell lane. Sequencing was performed using an Illumina HiSeq 2500 in “rapid run” mode employing a single-read, 50 base read length (SR50) sequencing strategy. Image analysis and base calling was performed using Illumina's Real Time Analysis v1.18 software, followed by 'demultiplexing' of indexed reads and generation of FASTQ files, using Illumina's bcl2fastq Conversion Software v1.8.4 (http://support.illumina.com/downloads/bcl2fastq_conversion_software_184.html). Reads of low quality were filtered prior to alignment to the reference genome (UCSC SacCer3 assembly) using TopHat v2.1.0[100]. Counts were generated from TopHat alignments for each gene using the Python package HTSeq v0.6.1[101]. Genes with low counts across all samples were removed, prior to identification of differentially expressed genes using the Bioconductor package edgeR v3.12.0[78]. A false discovery rate (FDR) method was employed to correct for multiple testing[102]. Differential expression was defined as |log_2_ (ratio) | ≥ 0.585 (± 1.5-fold) with the FDR set to 5%. Normalized differential expression data are available as excel files (S3 File), and raw data is available under NCBI GEO Series GSE69696.

### Comparison of transcriptional changes with *htz1Δ*

The overlap between lists of genes with significantly changed transcription compared to WT yeast in *htz1Δ* at t = 2 hours, and *psh1Δ pGAL-3Flag-CSE4* at t = 2 hours—t = 0 vs. WT t = 2 hours—t = 0 was found using the Whitehead Institute Compare Two Lists tool (http://jura.wi.mit.edu/bioc/tools/compare.php). The number of significantly up or down regulated transcripts overlapped between the genotypes was compared using the hypergeometric distribution (p-value is probability of getting more than the observed number of successes) using the total number of genes in the edgeR result files as the total population, using the GeneProf hypergeometric distribution calculator [103].

## Supporting Information

S1 Fig Characterization of ChIP-seq strains. (A) 5-fold serial dilutions of ChIP-seq strains (*3Flag-CSE4* (SBY10419), *psh1Δ 3Flag-CSE4* (SBY10484), *pGAL-3Flag-CSE4* (SBY10425) and *psh1Δ pGAL-3Flag-CSE4* (SBY10483)) grown on the indicated media at the indicated temperatures. (B) Immunoblot of CENP-A^Cse4^ and H2B levels in MNase-treated. chromatin (input) in the following strains: untagged WT (SBY3), *3Flag-CSE4* (SBY10419), *psh1Δ 3Flag-CSE4* (SBY10484), *pGAL-3Flag-CSE4* (SBY10425) and *psh1Δ pGAL-3Flag-CSE4* (SBY10483). (C) Immunoblot of samples from the ChIP-seq experiment showing 3Flag-Cse4 in the insoluble pellet (Pellet), the MNase-treated input chromatin (Input), the unbound material (after anti-FLAG IP) (Unbound), and the anti-FLAG IP for the following strains: *3Flag-CSE4* (SBY10419), *pGAL-3Flag-CSE4* (SBY10425), *psh1Δ 3Flag-CSE4* (SBY10484), and *psh1Δ pGAL-3Flag-CSE4* (SBY10483). Whole cell extract (WCE) from an untagged strain (SBY3) is also shown as a comparison. (D) Prepared Solexa libraries before sequencing for *3Flag-CSE4* (SBY10419), *pGAL-3Flag-CSE4* (SBY10425), *psh1Δ pGAL-3Flag-CSE4* (SBY10483) and *psh1Δ 3Flag-CSE4* (SBY10484), Both Input (IN) and IP are shown for each strain.
(TIF)

S2 Fig Validation of ChIP-seq data. (A) ChIP-qPCR validation of CENP-A^Cse4^ centromeric peaks at *CEN4*, the rDNA locus, *SAP4* promoter, *SLP1* promoter, and the *UTH1* gene. % Input (means +/- 1 SEM) is shown for 2–4 biological replicates. Strains used were: untagged WT (SBY3, black), *3Flag-CSE4* (SBY10419, blue), *psh1Δ 3Flag-CSE4* (SBY10484, pink), *pGAL-3Flag-CSE4* (SBY10425, green) and *psh1Δ pGAL-3Flag-CSE4* (SBY10483, orange). (B) Fraction of total centromere like regions (CLRs) or low confidence negative control regions (LCNCRs) [60] that overlap CENP-A^Cse4^ ChIP-seq peaks in each strain. (C-F) AT% per CENP-A^Cse4^ ChIP or input nucleosome peak for the *3Flag-CSE4* strain (SBY10419), the *psh1Δ 3Flag-CSE4* strain (SBY10484), the *pGAL-3Flag-CSE4* strain (SBY10425), or the *psh1Δ pGAL-3Flag-CSE4* strain (SBY10483).
(TIF)

S3 Fig Basal transcription levels are not correlated with CENP-A^Cse4^ mislocalization. (A-C) TSS and TTS profiles of CENP-A^Cse4^ ChIP compared to input nucleosome ChIP for *3Flag-CSE4* (SBY10419), *psh1Δ 3Flag-CSE4* (SBY10484), or *pGAL-3Flag-CSE4* (SBY10425) strains. Genes are binned by basal transcription level [61].
(TIF)

S4 Fig Transcription direction is correlated with CENP-A^Cse4^ mislocalization. (A-C) TSS and TTS profiles of CENP-A^Cse4^ ChIP for *3Flag-CSE4* (SBY10419), *psh1Δ 3Flag-CSE4* (SBY10484), or *pGAL-3Flag-CSE4* (SBY10425) strains. The 5’ and 3’ ends of genes are binned by direction of upstream or downstream transcription.
(TIF)

S5 Fig CENP-A^Cse4^ is mislocalized to nucleosomes flanking NDRs, origins of replication, and centromeres. (A-D) Mean CENP-A^Cse4^ ChIP coverage 500 bp upstream and downstream of all transcription start sites (TSS), for *3Flag-CSE4* (SBY10419), *psh1Δ 3Flag-CSE4* (SBY10484), *pGAL-3Flag-CSE4* (SBY10425), and *psh1Δ pGAL-3Flag-CSE4* (SBY10483) strains separated by the presence of an annotated NDR [65] within the promoter. (E) Mean CENP-A^Cse4^ ChIP coverage for the *psh1Δ pGAL-3Flag-CSE4* (SBY10483) strain (left y axis) vs. mean H2A.Z^Htz1^ ChIP coverage [4] (right y axis) at all origins of replication. (F) Mean CENP-A^Cse4^ ChIP coverage for *3Flag-CSE4* (SBY10419), *psh1Δ 3Flag-CSE4* (SBY10484), *pGAL-3Flag-CSE4* (SBY10425), *psh1Δ pGAL-3Flag-CSE4* (SBY10483) strains at all origins. (G) Mean CENP-A^Cse4^ ChIP coverage for *3Flag-CSE4* (SBY10419), *psh1Δ 3Flag-CSE4* (SBY10484), *pGAL-3Flag-CSE4* (SBY10425), and *psh1Δ pGAL-3Flag-CSE4* (SBY10483) strains at all centromeres.
(TIF)

S6 Fig CENP-A^Cse4^ localization to H2A.Z^Htz1^ nucleosomes. (A) Mean CENP-A^Cse4^ (from *psh1Δ pGAL-3Flag-CSE4* (SBY10483)) and H2A.Z^Htz1^ (from WT strain [4]) ChIP coverage 500 bp flanking all TSS. (B) Mean CENP-A^Cse4^ (from *psh1Δ pGAL-3Flag-CSE4* SBY10483) and H2A.Z^Htz1^ (from WT strain [4]) ChIP coverage 500 bp flanking all TTS. (C) CENP-A^Cse4^ ChIP coverage for the *3Flag-CSE4* (SBY10419, blue), *psh1Δ 3Flag-CSE4* (SBY10484, pink), *pGAL-3Flag-CSE4* (SBY10425, green), *psh1Δ pGAL-3Flag-CSE4* (SBY10483, orange) strain and WT H2A.Z^Htz1^ coverage [4] (blue) on the chromosome 4 arm between 60,000 bp and 130,000 bp. (D) Boxplot showing H2A.Z^Htz1^ score distributions for each H2A.Z^Htz1^ nucleosome with (black) or without (blue) overlapping CENP-A^Cse4^ peaks in the *3Flag-CSE4* (SBY10419), *psh1Δ pGAL-3Flag-CSE4* (SBY10484), *pGAL-3Flag-CSE4* (SBY10425) or *psh1Δ pGAL-3Flag-CSE4* (SBY10483) strains. (E) The percentage of CENP-A^Cse4^ peak centers separated by overlap with H2A.Z^Htz1^ peaks in each type of genomic region is graphed for the *psh1Δ pGAL-3Flag-CSE4* strain (SBY10483). See Fig 1B for the percentages of the total CENP-A^Cse4^ peaks in each genomic region.
(TIF)

S7 Fig The role of H2A.Z^Htz1^ and SWR-C in CENP-A^Cse4^ mislocalization. (A) Means +/- 1 SEM from quantitative immunoblots of chromatin fractionations measuring H2A.Z^Htz1^:H2B fold change vs. the *pGAL-3Flag-CSE4* strain. Strains used: *pGAL-3Flag-CSE4 3HA-HTZ1* (SBY12832), *swr1Δ pGAL-3Flag-CSE4 3HA-HTZ1* (SBY12956), *psh1Δ pGAL-3Flag-CSE4 3HA-HTZ1* (SBY12833), and *swr1Δ psh1Δ pGAL-3Flag-CSE4 3HA-HTZ1* (SBY12924). n = 3. (B) Immunoblot of a chromatin fractionation experiment from strains as in (A). Pgk1 is a marker of the soluble fraction and H2B is a marker of the chromatin fraction. (C) Stability assay of *pGAL-3Flag-CSE4* in WT (SBY12332), vs. *swr1Δ* (SBY12333) background. (-) samples were taken before adding 2% galactose to induce *pGAL-3Flag-CSE4* overexpression. 0–60 minute timepoints were taken after adding 2% glucose and cyclohexamide (50ug/ml) to inhibit transcription and translation of *pGAL-3Flag-CSE4*. Pgk1 is shown as a loading control. (D) HA-Htz1 ChIP in untagged WT (SBY3), *3HA-HTZ1 3Flag-CSE4* (SBY12918), *psh1Δ 3HA-HTZ1 3Flag-CSE4* (SBY13998), *3HA-HTZ1 pGAL-3Flag-CSE4* (SBY12832), *psh1Δ 3HA-HTZ1 pGAL-3Flag-CSE4* (SBY12833) at the *SAP4* and *RDS1* promoters and within the *ARE1* gene.
(TIF)

S8 Fig The role of INO80-C in CENP-A^Cse4^ localization. (A) Mean +/- 1 SEM from quantitative immunoblots of chromatin fractionations measuring H2A.Z^Htz1^:H2B fold change vs. *pGAL-3Flag-CSE4* strain. Strains used: *pGAL-3Flag-CSE4 HA-HTZ1* (SBY12832), *nhp10Δ pGAL-3Flag-CSE4 3HA-HTZ1* (SBY12930), *psh1Δ pGAL-3Flag-CSE4 3HA-HTZ1* (SBY12833), and *nhp10Δ psh1Δ pGAL-3Flag-CSE4 3HA-HTZ1* (SBY12959). n = 3. (B) Immunoblot of chromatin fractionation from the strains in (A). Pgk1 is a marker of the soluble fraction and H2B is a marker of the chromatin fraction. (C) 5-fold serial dilutions of WT (SBY3939), *nhp10Δ* (SBY11577), *psh1Δ* (SBY8336), *psh1Δ nhp10Δ* (SBY12346), *pGAL-3Flag-CSE4* (SBY12349), *nhp10Δ pGAL-3Flag-CSE4* (SBY12317), *psh1Δ pGAL-3Flag-CSE4* (SBY12350), *nhp10Δ psh1Δ pGAL-3Flag-CSE4* (SBY12348) strains on indicated media. (D) Immunoblot of CENP-A^Cse4^ levels when induced with low levels of galactose (0.1%) in *psh1Δ pGAL-3Flag-CSE4 3HA-HTZ1* (SBY12833) and *nhp10Δ psh1Δ pGAL-3Flag-CSE4 3HA-HTZ1* (SBY12959) cells. Pgk1 is shown as a loading control. (E) Stability assay of *pGAL-3Flag-CSE4* in wild-type (SBY12349) vs. *nhp10Δ* (SBY12317) background. (-) samples were taken before adding 2% galactose to induce *pGAL-3Flag-CSE4* overexpression. 0–60 minute timepoints were taken after adding 2% glucose and cyclohexamide (50ug/ml) to stop transcription and translation of *pGAL-3Flag-CSE4*. Pgk1 is shown as a loading control.
(TIF)

S9 Fig Comparison of CENP-A^Cse4^ promoter mislocalization and changes in gene expression. (A) rDNA copy number ratio comparing the rDNA to *UTH1*, a single copy gene. Graph shows the mean ratio +/- 1 SEM for 3 biological replicates. Strains used were: *3Flag-CSE4* (SBY10419), *psh1Δ 3Flag-CSE4* (SBY10484), *pGAL-3Flag-CSE4* (SBY10425), *psh1Δ pGAL-3Flag-CSE4* (SBY10425). (B) rRNA expression analysis compared to *ACT1* at t0 and t2 for strains used in the RNA-seq experiment (see S5 Table). Graph shows mean ratio +/- 1 standard deviation. (n = 2–3) (C) Cell cycle distribution of cell cycle regulated genes differentially expressed (DE) at t2 in *psh1Δ pGAL-3Flag-CSE4*. (D) Proportional Venn diagram of genes upregulated in *psh1Δ pGAL-3Flag-CSE4* at t = 2 hours and genes with CENP-A^Cse4^ peaks in their promoters in the *psh1Δ pGAL-3Flag-CSE4* strain. p = 0.009 from a cumulative hypergeometric distribution test. (E) TSS and TTS profiles of CENP-A^Cse4^ ChIP coverage in the *psh1Δ pGAL-3Flag-CSE4* (SBY10483) strain at all genes (blue), upregulated differentially expressed (DE) genes in *psh1Δ pGAL-3Flag-CSE4* only (red), upregulated DE genes in *htz1Δ* only (green), or upregulated DE genes in both *psh1Δ pGAL-3Flag-CSE4* and *htz1Δ* (purple).
(TIF)

S1 Table ChIP-seq information. (PDF)

S2 Table Yeast strains used in this study. (PDF)

S3 Table Plasmids used in this study. (PDF)

S4 Table Oligonucleotides used in this study. (PDF)

S5 Table RNA-seq information. (PDF)

S1 File RNA-seq differential gene expression data. (XLSX)

S2 File Transcription factor enrichment analysis. (XLSX)

S3 File Supplemental methods. (PDF)
