# Supplementary figures and images for: Regulation of Budding Yeast CENP-A levels Prevents Misincorporation at Promoter Nucleosomes and Transcriptional Defects
Source: PLoS Genet. 2016 Mar 16;12(3):e1005930. doi: 10.1371/journal.pgen.1005930 (PMC4794243; doi:10.1371/journal.pgen.1005930)

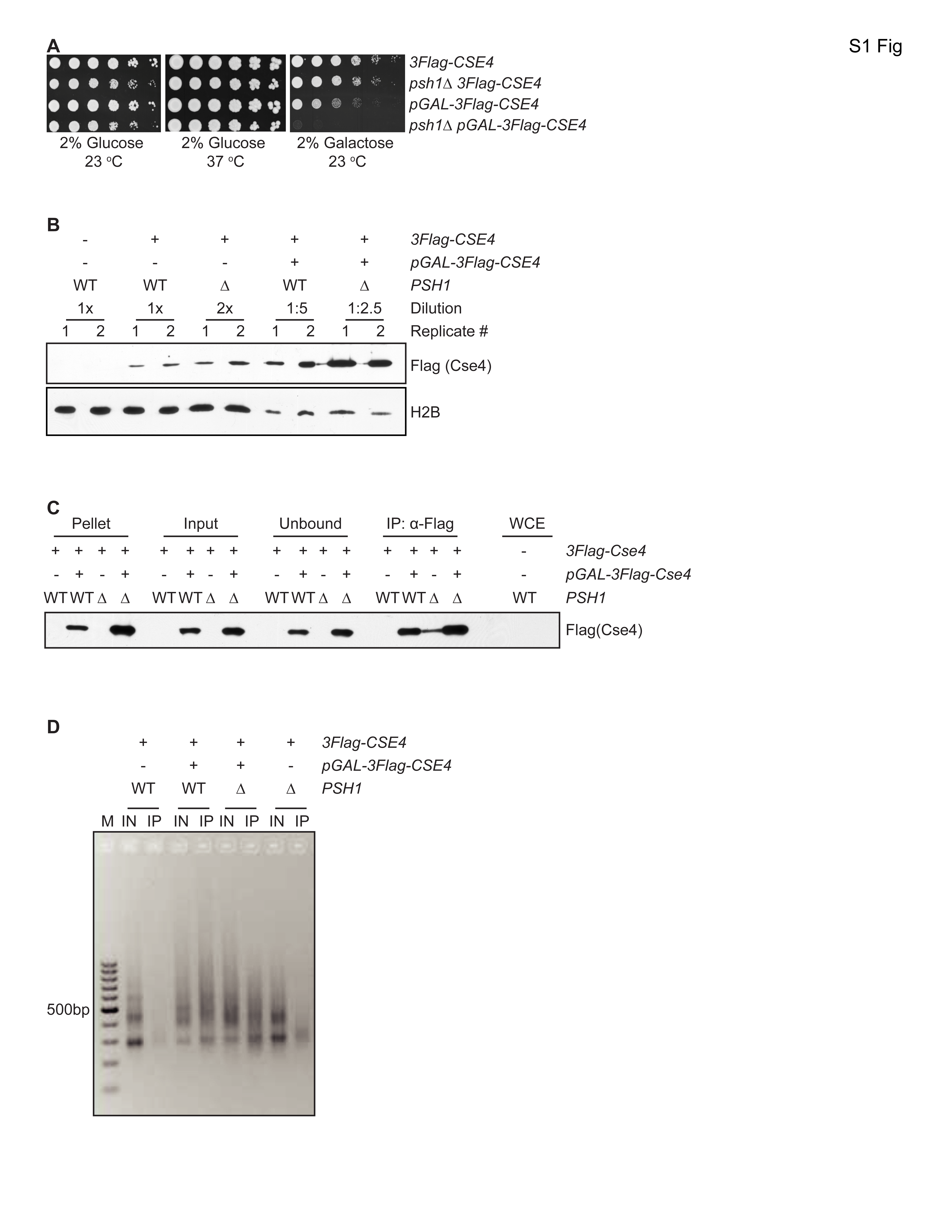

Supplement: S1 Fig — (A) 5-fold serial dilutions of ChIP-seq strains (3Flag-CSE4 (SBY10419), psh1Δ 3Flag-CSE4 (SBY10484), pGAL-3Flag-CSE4 (SBY10425) and psh1Δ pGAL-3Flag-CSE4 (SBY10483)) grown on the indicated media at the indicated temperatures. (B) Immunoblot of CENP-ACse4 and H2B levels in MNase-treated. chromatin (input) in the following strains: untagged WT (SBY3), 3Flag-CSE4 (SBY10419), psh1Δ 3Flag-CSE4 (SBY10484), pGAL-3Flag-CSE4 (SBY10425) and psh1Δ pGAL-3Flag-CSE4 (SBY10483). (C) Immunoblot of samples from the ChIP-seq experiment showing 3Flag-Cse4 in the insoluble pellet (Pellet), the MNase-treated input chromatin (Input), the unbound material (after anti-FLAG IP) (Unbound), and the anti-FLAG IP for the following strains: 3Flag-CSE4 (SBY10419), pGAL-3Flag-CSE4 (SBY10425), psh1Δ 3Flag-CSE4 (SBY10484), and psh1Δ pGAL-3Flag-CSE4 (SBY10483). Whole cell extract (WCE) from an untagged strain (SBY3) is also shown as a comparison. (D) Prepared Solexa libraries before sequencing for 3Flag-CSE4 (SBY10419), pGAL-3Flag-CSE4 (SBY10425), psh1Δ pGAL-3Flag-CSE4 (SBY10483) and psh1Δ 3Flag-CSE4 (SBY10484), Both Input (IN) and IP are shown for each strain. (TIF) [file pgen.1005930.s001.tif]

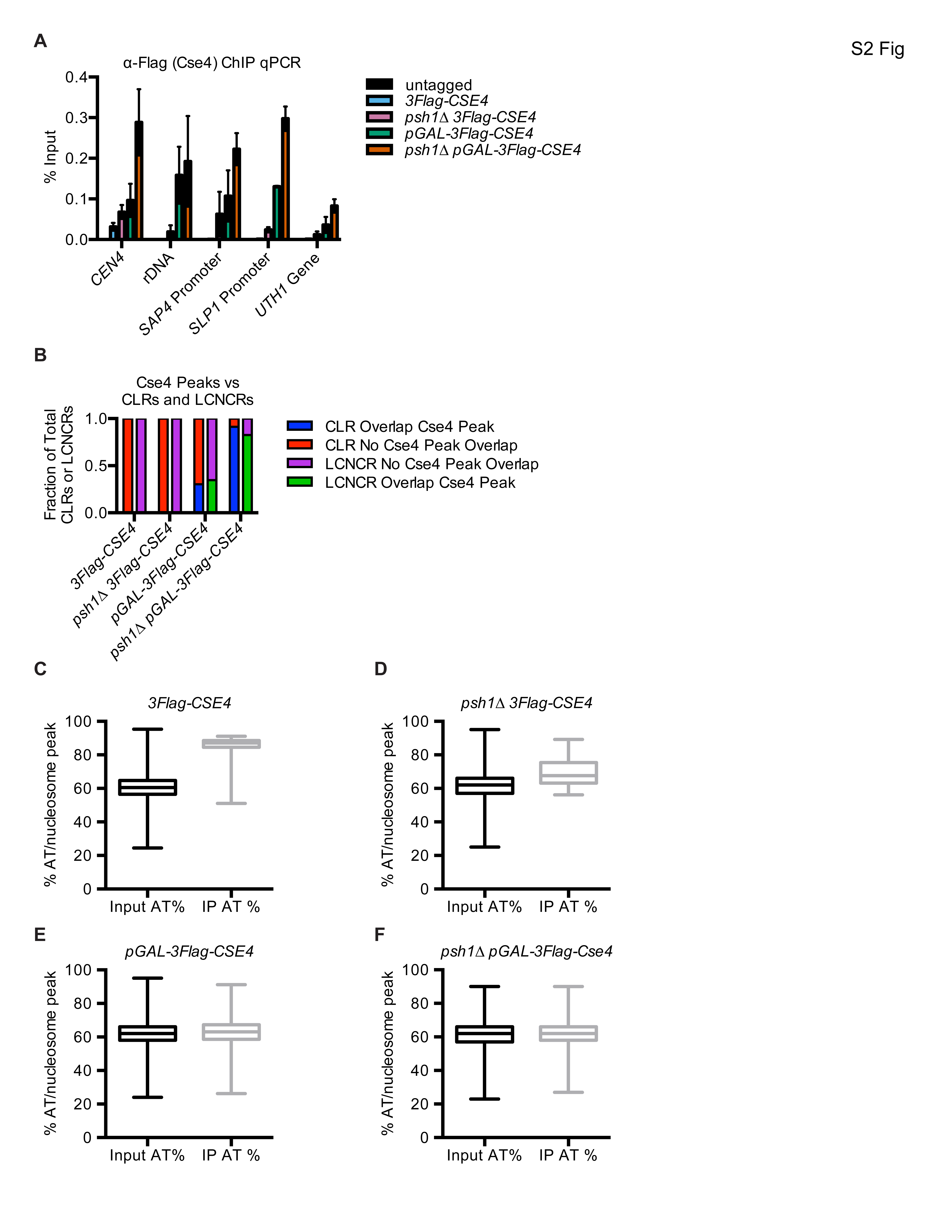

Supplement: S2 Fig — (A) ChIP-qPCR validation of CENP-ACse4 centromeric peaks at CEN4, the rDNA locus, SAP4 promoter, SLP1 promoter, and the UTH1 gene. % Input (means +/- 1 SEM) is shown for 2–4 biological replicates. Strains used were: untagged WT (SBY3, black), 3Flag-CSE4 (SBY10419, blue), psh1Δ 3Flag-CSE4 (SBY10484, pink), pGAL-3Flag-CSE4 (SBY10425, green) and psh1Δ pGAL-3Flag-CSE4 (SBY10483, orange). (B) Fraction of total centromere like regions (CLRs) or low confidence negative control regions (LCNCRs) [60] that overlap CENP-ACse4 ChIP-seq peaks in each strain. (C-F) AT% per CENP-ACse4 ChIP or input nucleosome peak for the 3Flag-CSE4 strain (SBY10419), the psh1Δ 3Flag-CSE4 strain (SBY10484), the pGAL-3Flag-CSE4 strain (SBY10425), or the psh1Δ pGAL-3Flag-CSE4 strain (SBY10483). (TIF) [file pgen.1005930.s002.tif]

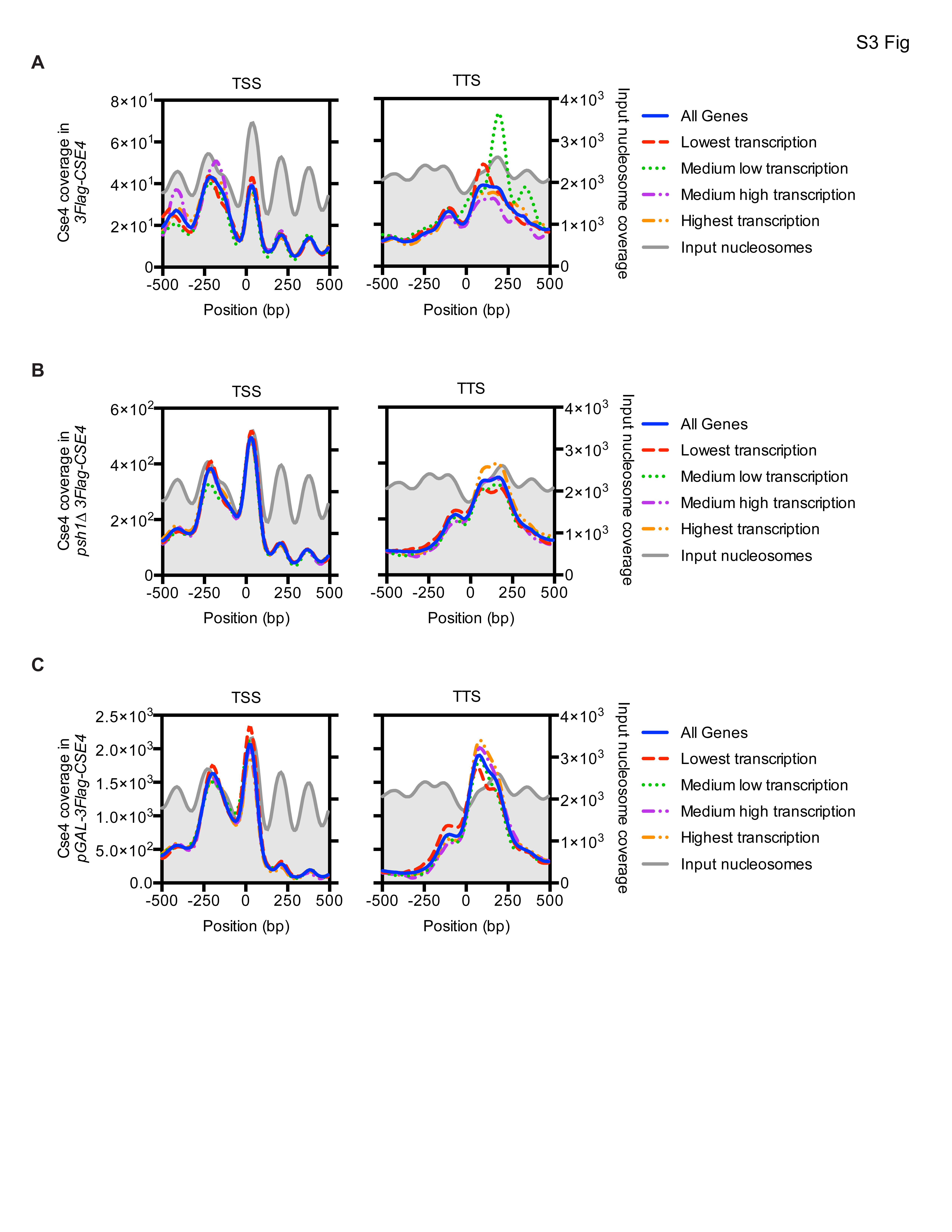

Supplement: S3 Fig — (A-C) TSS and TTS profiles of CENP-ACse4 ChIP compared to input nucleosome ChIP for 3Flag-CSE4 (SBY10419), psh1Δ 3Flag-CSE4 (SBY10484), or pGAL-3Flag-CSE4 (SBY10425) strains. Genes are binned by basal transcription level [61]. (TIF) [file pgen.1005930.s003.tif]

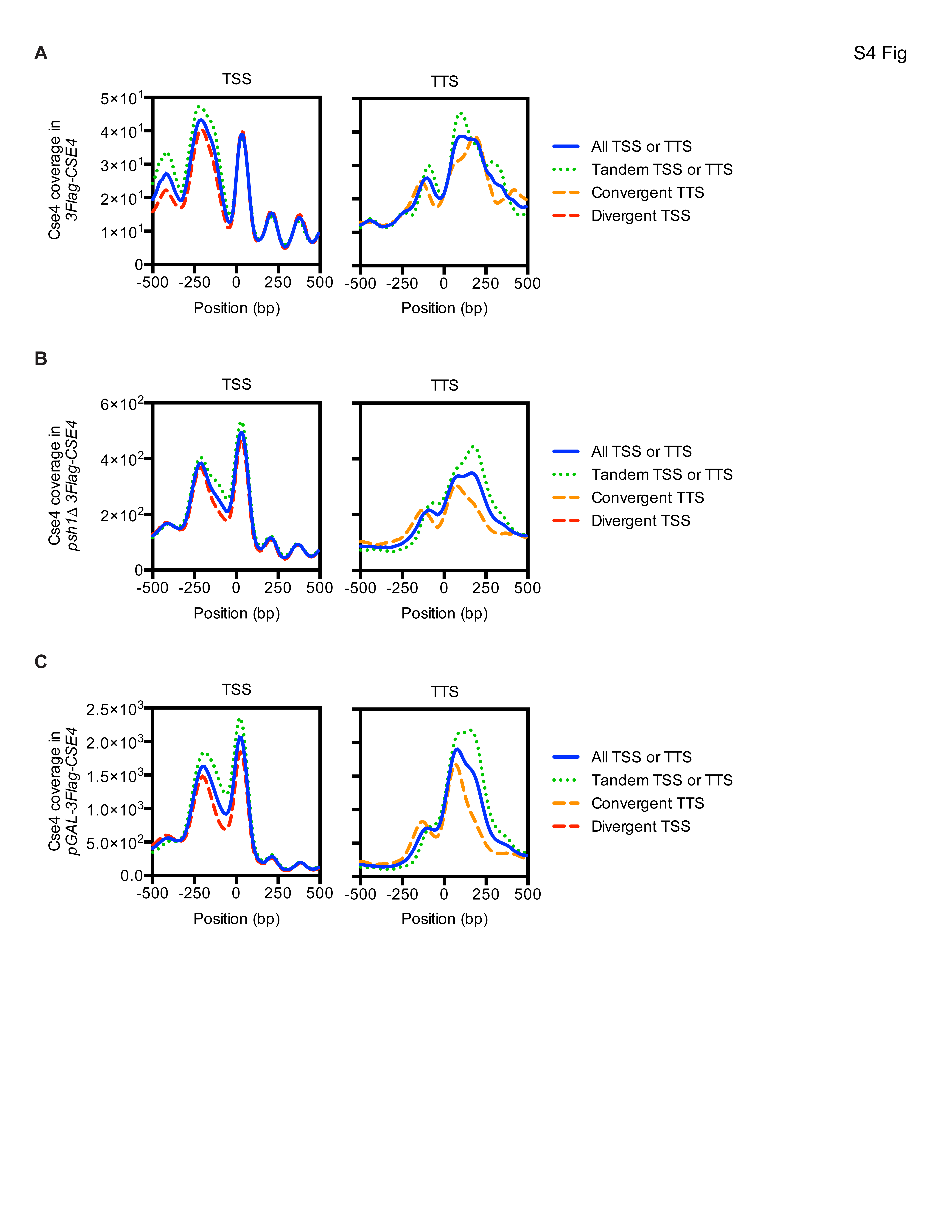

Supplement: S4 Fig — (A-C) TSS and TTS profiles of CENP-ACse4 ChIP for 3Flag-CSE4 (SBY10419), psh1Δ 3Flag-CSE4 (SBY10484), or pGAL-3Flag-CSE4 (SBY10425) strains. The 5’ and 3’ ends of genes are binned by direction of upstream or downstream transcription. (TIF) [file pgen.1005930.s004.tif]

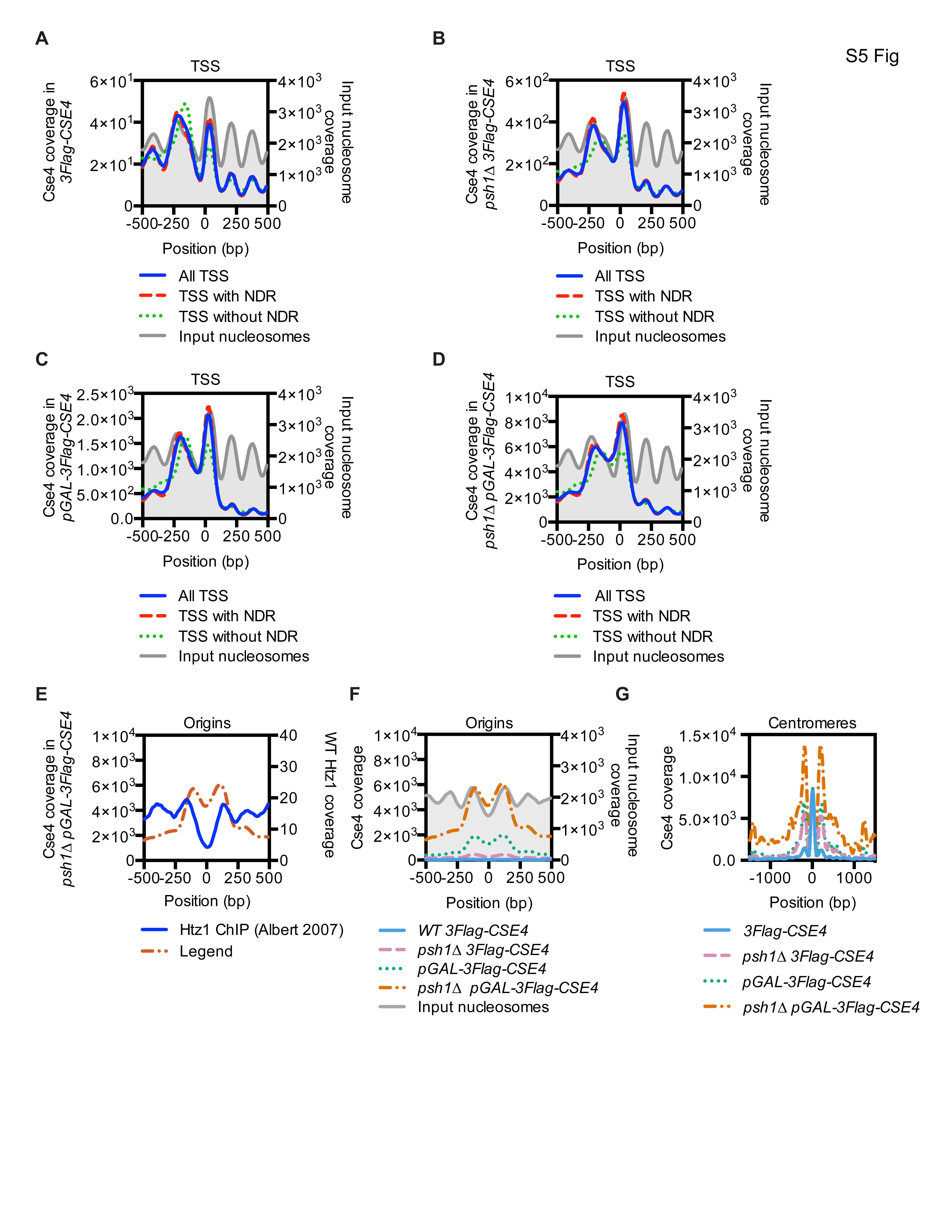

Supplement: S5 Fig — (A-D) Mean CENP-ACse4 ChIP coverage 500 bp upstream and downstream of all transcription start sites (TSS), for 3Flag-CSE4 (SBY10419), psh1Δ 3Flag-CSE4 (SBY10484), pGAL-3Flag-CSE4 (SBY10425), and psh1Δ pGAL-3Flag-CSE4 (SBY10483) strains separated by the presence of an annotated NDR [65] within the promoter. (E) Mean CENP-ACse4 ChIP coverage for the psh1Δ pGAL-3Flag-CSE4 (SBY10483) strain (left y axis) vs. mean H2A.ZHtz1 ChIP coverage [4] (right y axis) at all origins of replication. (F) Mean CENP-ACse4 ChIP coverage for 3Flag-CSE4 (SBY10419), psh1Δ 3Flag-CSE4 (SBY10484), pGAL-3Flag-CSE4 (SBY10425), psh1Δ pGAL-3Flag-CSE4 (SBY10483) strains at all origins. (G) Mean CENP-ACse4 ChIP coverage for 3Flag-CSE4 (SBY10419), psh1Δ 3Flag-CSE4 (SBY10484), pGAL-3Flag-CSE4 (SBY10425), and psh1Δ pGAL-3Flag-CSE4 (SBY10483) strains at all centromeres. (TIF) [file pgen.1005930.s005.tif]

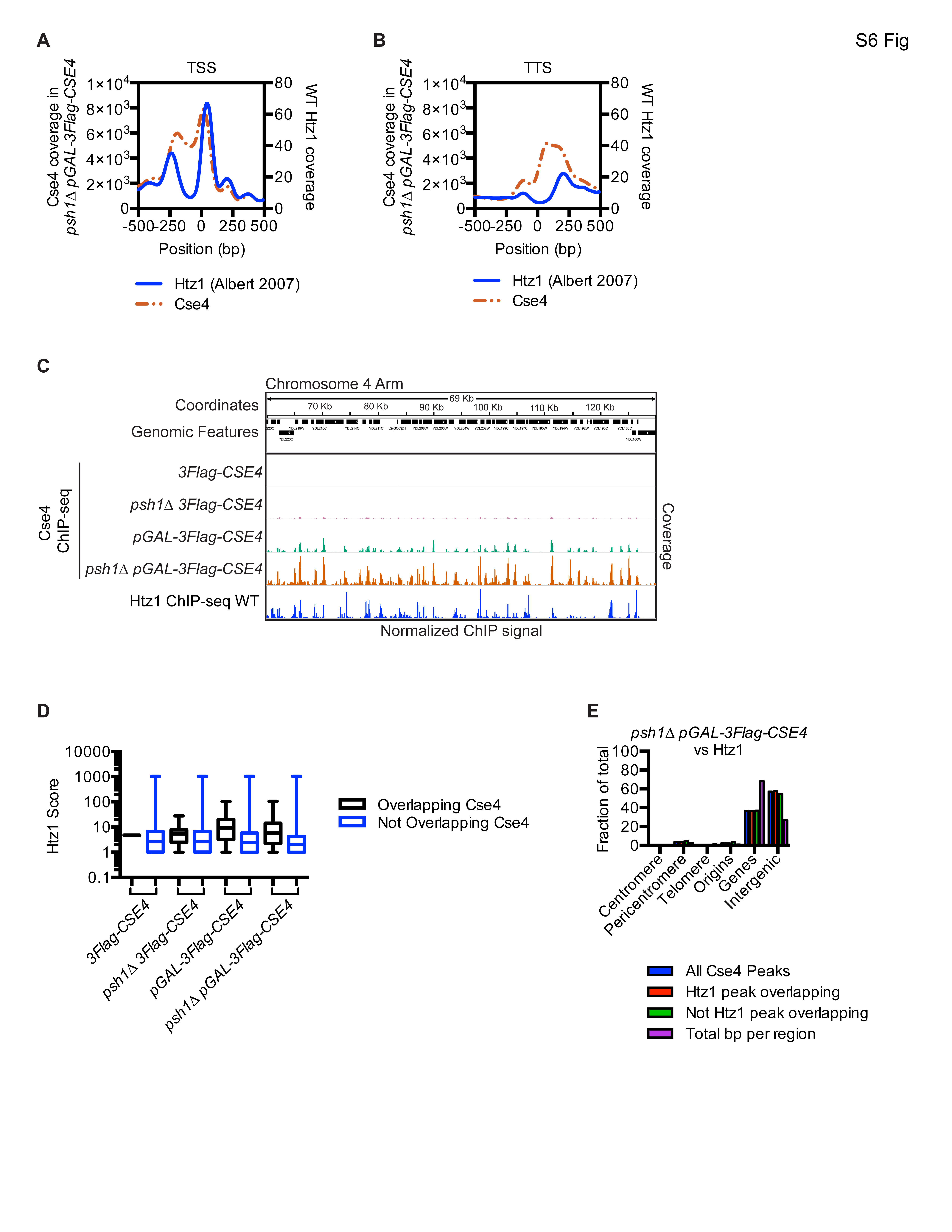

Supplement: S6 Fig — (A) Mean CENP-ACse4 (from psh1Δ pGAL-3Flag-CSE4 (SBY10483)) and H2A.ZHtz1 (from WT strain [4]) ChIP coverage 500 bp flanking all TSS. (B) Mean CENP-ACse4 (from psh1Δ pGAL-3Flag-CSE4 SBY10483) and H2A.ZHtz1 (from WT strain [4]) ChIP coverage 500 bp flanking all TTS. (C) CENP-ACse4 ChIP coverage for the 3Flag-CSE4 (SBY10419, blue), psh1Δ 3Flag-CSE4 (SBY10484, pink), pGAL-3Flag-CSE4 (SBY10425, green), psh1Δ pGAL-3Flag-CSE4 (SBY10483, orange) strain and WT H2A.ZHtz1 coverage [4] (blue) on the chromosome 4 arm between 60,000 bp and 130,000 bp. (D) Boxplot showing H2A.ZHtz1 score distributions for each H2A.ZHtz1 nucleosome with (black) or without (blue) overlapping CENP-ACse4 peaks in the 3Flag-CSE4 (SBY10419), psh1Δ pGAL-3Flag-CSE4 (SBY10484), pGAL-3Flag-CSE4 (SBY10425) or psh1Δ pGAL-3Flag-CSE4 (SBY10483) strains. (E) The percentage of CENP-ACse4 peak centers separated by overlap with H2A.ZHtz1 peaks in each type of genomic region is graphed for the psh1Δ pGAL-3Flag-CSE4 strain (SBY10483). See Fig 1B for the percentages of the total CENP-ACse4 peaks in each genomic region. (TIF) [file pgen.1005930.s006.tif]

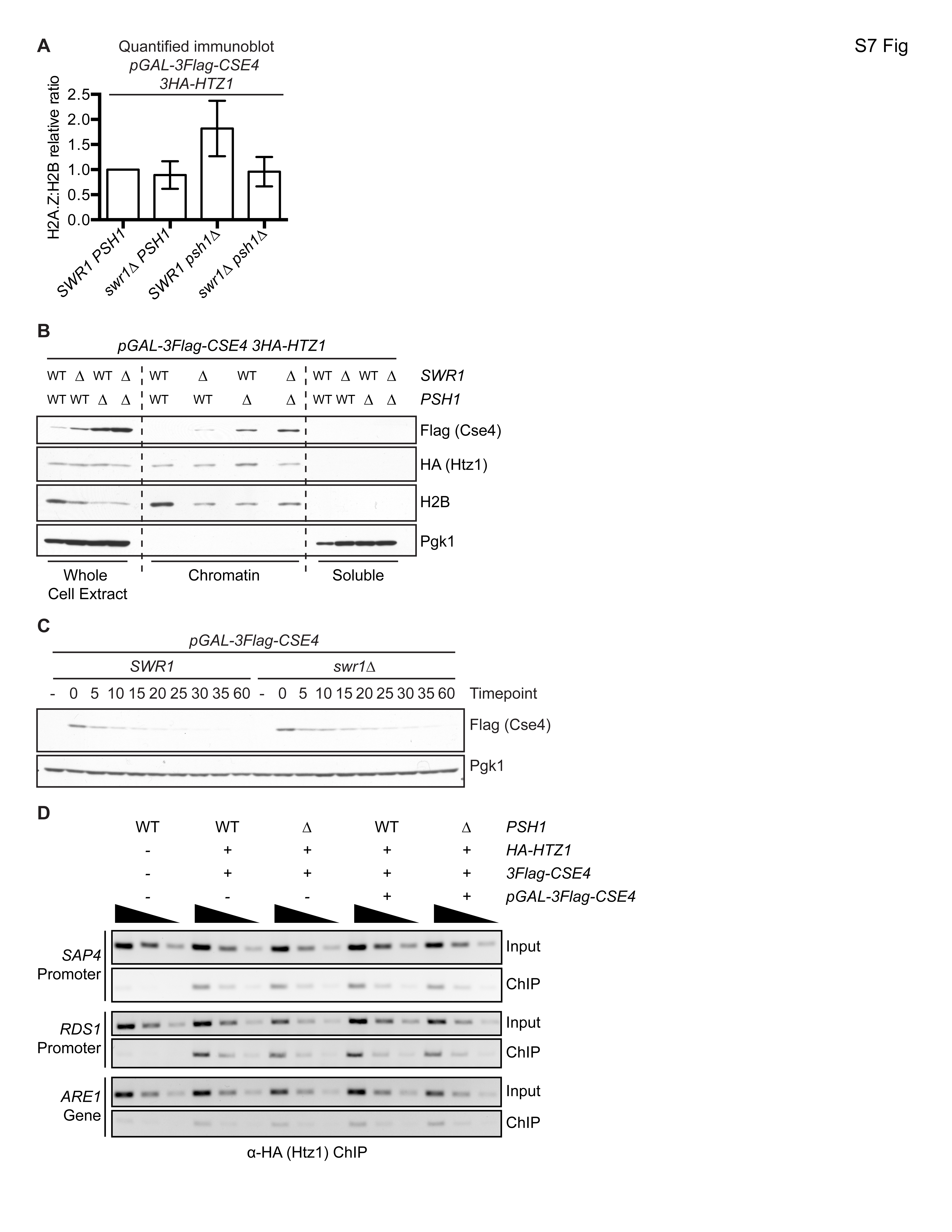

Supplement: S7 Fig — (A) Means +/- 1 SEM from quantitative immunoblots of chromatin fractionations measuring H2A.ZHtz1:H2B fold change vs. the pGAL-3Flag-CSE4 strain. Strains used: pGAL-3Flag-CSE4 3HA-HTZ1 (SBY12832), swr1Δ pGAL-3Flag-CSE4 3HA-HTZ1 (SBY12956), psh1Δ pGAL-3Flag-CSE4 3HA-HTZ1 (SBY12833), and swr1Δ psh1Δ pGAL-3Flag-CSE4 3HA-HTZ1 (SBY12924). n = 3. (B) Immunoblot of a chromatin fractionation experiment from strains as in (A). Pgk1 is a marker of the soluble fraction and H2B is a marker of the chromatin fraction. (C) Stability assay of pGAL-3Flag-CSE4 in WT (SBY12332), vs. swr1Δ (SBY12333) background. (-) samples were taken before adding 2% galactose to induce pGAL-3Flag-CSE4 overexpression. 0–60 minute timepoints were taken after adding 2% glucose and cyclohexamide (50ug/ml) to inhibit transcription and translation of pGAL-3Flag-CSE4. Pgk1 is shown as a loading control. (D) HA-Htz1 ChIP in untagged WT (SBY3), 3HA-HTZ1 3Flag-CSE4 (SBY12918), psh1Δ 3HA-HTZ1 3Flag-CSE4 (SBY13998), 3HA-HTZ1 pGAL-3Flag-CSE4 (SBY12832), psh1Δ 3HA-HTZ1 pGAL-3Flag-CSE4 (SBY12833) at the SAP4 and RDS1 promoters and within the ARE1 gene. (TIF) [file pgen.1005930.s007.tif]

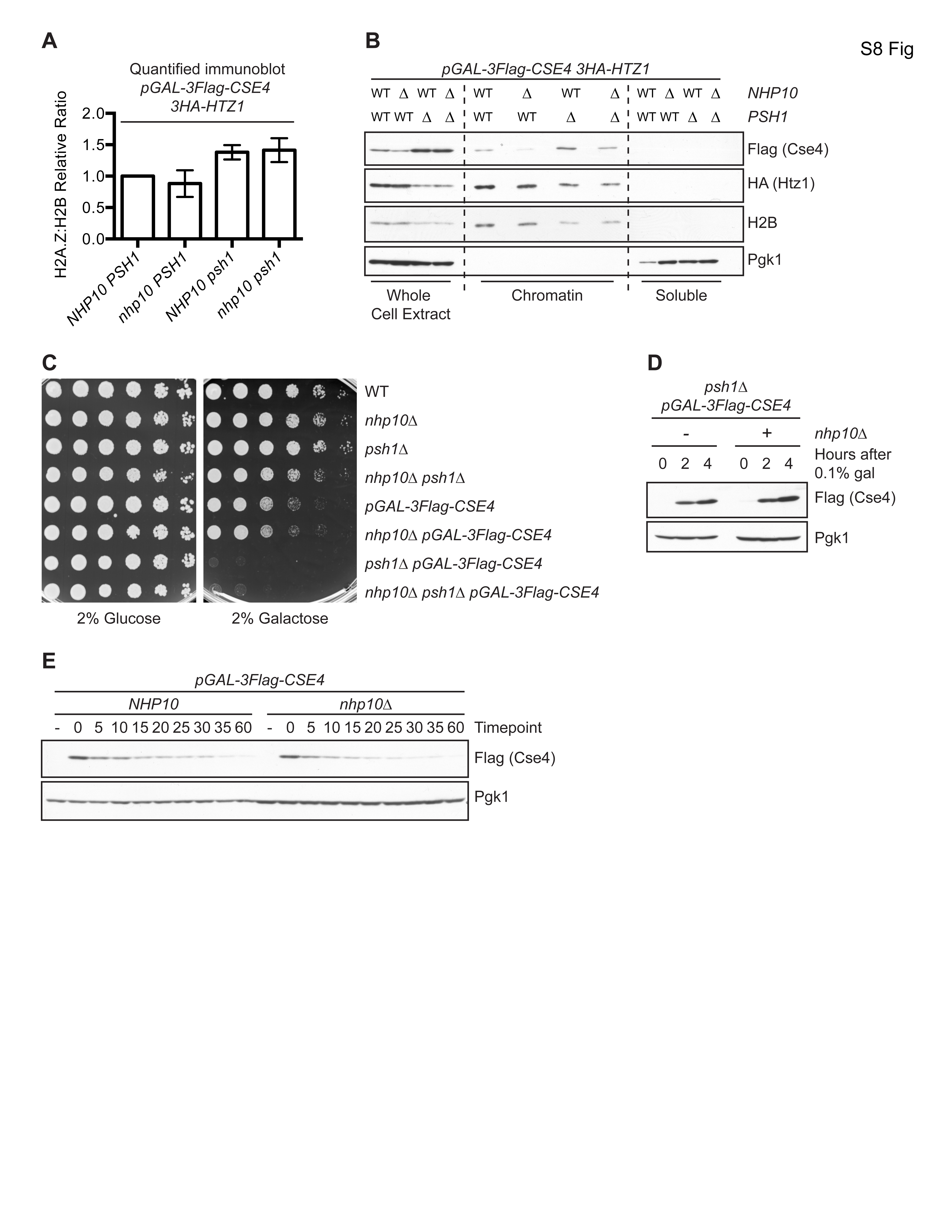

Supplement: S8 Fig — (A) Mean +/- 1 SEM from quantitative immunoblots of chromatin fractionations measuring H2A.ZHtz1:H2B fold change vs. pGAL-3Flag-CSE4 strain. Strains used: pGAL-3Flag-CSE4 HA-HTZ1 (SBY12832), nhp10Δ pGAL-3Flag-CSE4 3HA-HTZ1 (SBY12930), psh1Δ pGAL-3Flag-CSE4 3HA-HTZ1 (SBY12833), and nhp10Δ psh1Δ pGAL-3Flag-CSE4 3HA-HTZ1 (SBY12959). n = 3. (B) Immunoblot of chromatin fractionation from the strains in (A). Pgk1 is a marker of the soluble fraction and H2B is a marker of the chromatin fraction. (C) 5-fold serial dilutions of WT (SBY3939), nhp10Δ (SBY11577), psh1Δ (SBY8336), psh1Δ nhp10Δ (SBY12346), pGAL-3Flag-CSE4 (SBY12349), nhp10Δ pGAL-3Flag-CSE4 (SBY12317), psh1Δ pGAL-3Flag-CSE4 (SBY12350), nhp10Δ psh1Δ pGAL-3Flag-CSE4 (SBY12348) strains on indicated media. (D) Immunoblot of CENP-ACse4 levels when induced with low levels of galactose (0.1%) in psh1Δ pGAL-3Flag-CSE4 3HA-HTZ1 (SBY12833) and nhp10Δ psh1Δ pGAL-3Flag-CSE4 3HA-HTZ1 (SBY12959) cells. Pgk1 is shown as a loading control. (E) Stability assay of pGAL-3Flag-CSE4 in wild-type (SBY12349) vs. nhp10Δ (SBY12317) background. (-) samples were taken before adding 2% galactose to induce pGAL-3Flag-CSE4 overexpression. 0–60 minute timepoints were taken after adding 2% glucose and cyclohexamide (50ug/ml) to stop transcription and translation of pGAL-3Flag-CSE4. Pgk1 is shown as a loading control. (TIF) [file pgen.1005930.s008.tif]

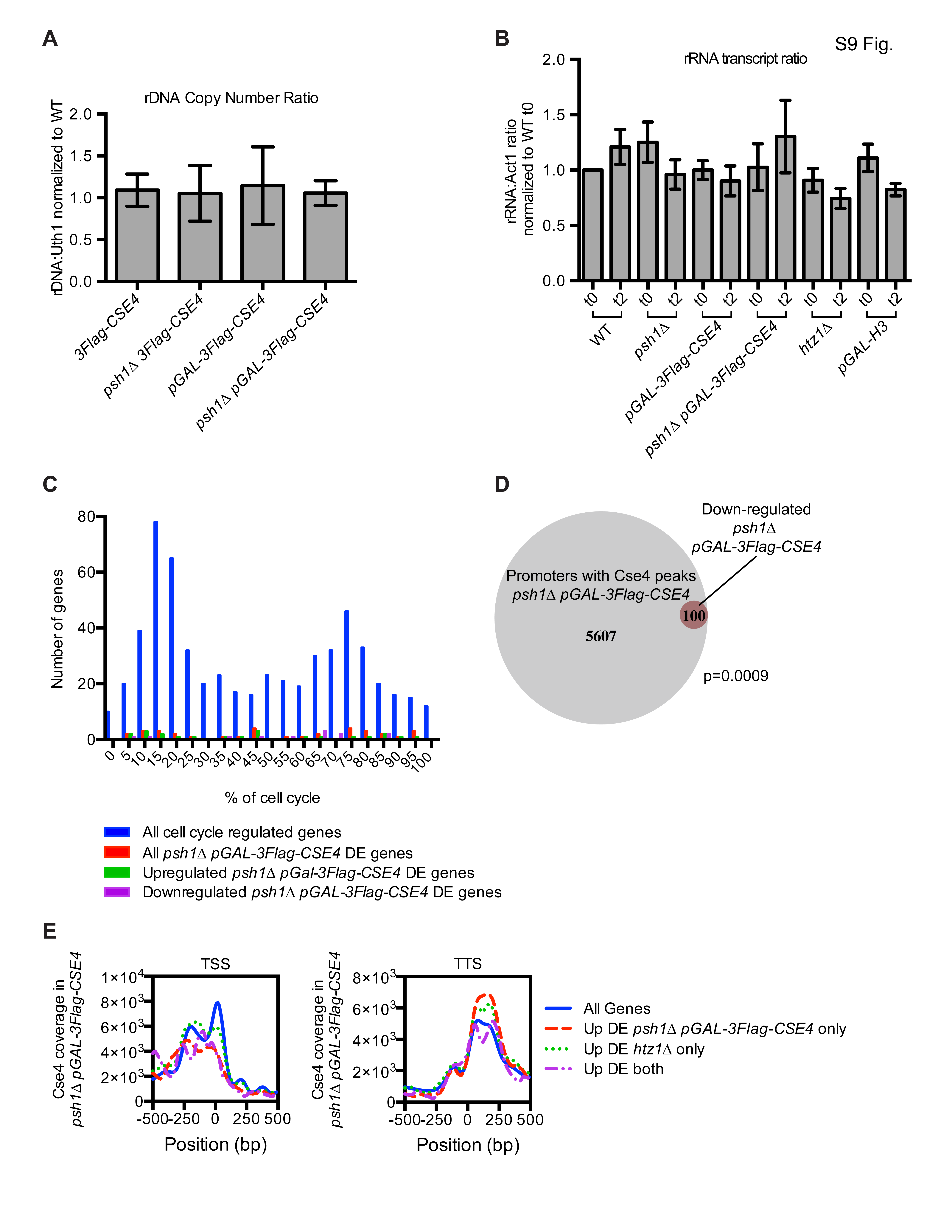

Supplement: S9 Fig — (A) rDNA copy number ratio comparing the rDNA to UTH1, a single copy gene. Graph shows the mean ratio +/- 1 SEM for 3 biological replicates. Strains used were: 3Flag-CSE4 (SBY10419), psh1Δ 3Flag-CSE4 (SBY10484), pGAL-3Flag-CSE4 (SBY10425), psh1Δ pGAL-3Flag-CSE4 (SBY10425). (B) rRNA expression analysis compared to ACT1 at t0 and t2 for strains used in the RNA-seq experiment (see S5 Table). Graph shows mean ratio +/- 1 standard deviation. (n = 2–3) (C) Cell cycle distribution of cell cycle regulated genes differentially expressed (DE) at t2 in psh1Δ pGAL-3Flag-CSE4. (D) Proportional Venn diagram of genes upregulated in psh1Δ pGAL-3Flag-CSE4 at t = 2 hours and genes with CENP-ACse4 peaks in their promoters in the psh1Δ pGAL-3Flag-CSE4 strain. p = 0.009 from a cumulative hypergeometric distribution test. (E) TSS and TTS profiles of CENP-ACse4 ChIP coverage in the psh1Δ pGAL-3Flag-CSE4 (SBY10483) strain at all genes (blue), upregulated differentially expressed (DE) genes in psh1Δ pGAL-3Flag-CSE4 only (red), upregulated DE genes in htz1Δ only (green), or upregulated DE genes in both psh1Δ pGAL-3Flag-CSE4 and htz1Δ (purple). (TIF) [file pgen.1005930.s009.tif]
