# Supplementary material for: Regulation of Budding Yeast CENP-A levels Prevents Misincorporation at Promoter Nucleosomes and Transcriptional Defects
Source: PLoS Genet. 2016 Mar 16;12(3):e1005930. doi: 10.1371/journal.pgen.1005930 (PMC4794243; doi:10.1371/journal.pgen.1005930)
Supplement: S1 Table — (PDF) [file pgen.1005930.s010.pdf]

S1 Table: ChIP-seq information

Strain	Input Correctly Mapped Mono-nucleosomal Read #	IP Correctly Mapped Mono-nucleosomal Read #	Input Average Read Length	IP Average Read Length	Average Mono-nucleosomal Read Depth/bp Input	Average Mono-nucleosomal Read Depth/bp IP
3Flag-CSE4 (SBY10419)	22462364	3016017	164	161	303	203
psh1Δ 3Flag-CSE4 (SBY10484)	19476982	1530412	148	147	238	88
pGAL-3Flag-CSE4 (SBY10425)	54398610	17742746	158	158	708	1145
psh1Δ pGAL-3Flag-CSE4 (SBY10483)	37815050	13220125	149	154	464	602
