# Supplementary material for: Regulation of Budding Yeast CENP-A levels Prevents Misincorporation at Promoter Nucleosomes and Transcriptional Defects
Source: PLoS Genet. 2016 Mar 16;12(3):e1005930. doi: 10.1371/journal.pgen.1005930 (PMC4794243; doi:10.1371/journal.pgen.1005930)
Supplement: S2 Table — (PDF) [file pgen.1005930.s011.pdf]

S2 Table. Yeast Strains used in this study.

All strains are isogenic to W303

Strain	Genotype	Integrated and [replicating] plasmids	Source
SBY3	*MAT**a** ura3-1 leu2,3-112 his3-11 trp1-1 ade2-1 can1-100 bar1-1 bud4 rad5-535*		
SBY3939	*MAT**a** ura3-1 leu2,3-112 his3-11 trp1-1 ade2-1 can1-100 bar1-1 bud4 RAD5*		
SBY4471	*MAT**a** ura3-1 leu2,3-112 his3-11 trp1-1::pGAL-H3:TRP1 ade2-1 can1-100 bar1-1 bud4 RAD5*	*pSB893*	
SBY6183	*MAT**a** ura3-1 leu2,3-112 his3-11 trp1-1 ade2-1 can1-100 bar1-1 bud4 RAD5 htz1::HYG*		[1]
SBY9540	*MAT**a** ura3-1 leu2,3-112::pGAL-3Flag-CSE4::LEU2 his3-11 trp1-1 ade2-1 can1-100 bar1-1 bud4 rad5-535*	*pSB1729*	
SBY10419	*MAT**a** ura3-1::pCSE4-3Flag-CSE4::URA leu2,3-112 his3-11 trp1-1 ade2-1 can1-100 bar1-1 bud4 RAD5 cse4::KAN*	*pSB1067*	
SBY10425	*MAT**a** ura3-1::pCSE4-3Flag-CSE4::URA leu2,3-112::pGAL-3Flag-CSE4::LEU2 his3-11 trp1-1 ade2-1 can1-100 bar1-1 bud4 RAD5 cse4::KAN*	*pSB1067, pSB1729*	
SBY10483	*MAT**a** ura3-1::pCSE4-3Flag-CSE4::URA leu2,3-112::pGAL-3Flag-CSE4::LEU2 his3-11 trp1-1*	*pSB1067, pSB1729*	

	ade2-1 can1-100 bar1-1 bud4 RAD5 cse4::KAN psh1::KAN		
SBY10484	*MAT**a** ura3-1::pCSE4-3Flag-CSE4::URA leu2,3-112 his3-11 trp1-1 ade2-1 can1-100 bar1-1 bud4 RAD5 cse4::KAN psh1::KAN*	*pSB1067*	
SBY12317	*MAT**a** ura3-1 leu2,3-112::pGAL-3Flag-CSE4::LEU2 his3-11 trp1-1 ade2-1 can1-100 bar1-1 bud4 rad5-535 nhp10::HYG*	*pSB1729*	*nhp10::HYG* from [2]
SBY12332	*MAT**a** ura3-1 leu2,3-112::pGAL-3Flag-CSE4::LEU2 his3-11 trp1-1 ade2-1 can1-100 bar1-1 bud4 rad5-535*	*pSB1729*	
SBY12333	*MAT**a** ura3-1 leu2,3-112::pGAL-3Flag-CSE4::LEU2 his3-11 trp1-1 ade2-1 can1-100 bar1-1 bud4 rad5-535 swr1::HYG*	*pSB1729*	*swr1::HYG* from [3]
SBY12338	*MAT**a** ura3-1 leu2,3-112::pGAL-3Flag-CSE4::LEU2 his3-11 trp1-1 ade2-1 can1-100 bar1-1 bud4 RAD5 psh1::KAN*	*pSB1729*	
SBY12349	*MAT**a** ura3-1 leu2,3-112::pGAL-3Flag-CSE4::LEU2 his3-11 trp1-1 ade2-1 can1-100 bar1-1 bud4 RAD5*	*pSB1729*	
SBY12350	*MAT**a** ura3-1 leu2,3-112::pGAL-3Flag-CSE4::LEU2 his3-11 trp1-1 ade2-1 can1-100 bar1-1 bud4 RAD5 psh1::KAN*	*pSB1729*	

SBY12779	*MAT**a** ura3-1 leu2,3-112 his3-11 trp1-1 ade2-1 can1-100 bar1-1 bud5 RAD5 htz1::HYG::pHTZ1-3HA-HTZ1::TRP*	*pSB1072*
SBY12832	*MAT**a** ura3-1::pCSE4-3Flag-CSE4::URA leu2,3-112::pGAL-3Flag-CSE4::LEU2 his3-11 trp1-1 ade2-1 can1-100 bar1-1 bud4 RAD5 htz1::HYG::pHTZ1-3HA-HTZ1::TRP cse4::KAN*	*pSB1067, pSB1072, pSB1729*
SBY12833	*MAT**a** ura3-1::pCSE4-3Flag-CSE4::URA leu2,3-112::pGAL-3Flag-CSE4::LEU2 his3-11 trp1-1 ade2-1 can1-100 bar1-1 bud4 RAD5 htz1::HYG::pHTZ1-3HA-HTZ1::TRP cse4::KAN psh1::KAN*	*pSB1067, pSB1072, pSB1729*
SBY12918	*MAT**a** ura3-1::pCSE4-3Flag-CSE4::URA leu2,3-112 his3-11 trp1-1 ade2-1 can1-100 bar1-1 bud4 RAD5 htz1::HYG::pHTZ1-3HA-HTZ1::TRP cse4::KAN*	*pSB1067, pSB1072*
SBY12920	*MAT**a** ura3-1::pCSE4-3Flag-CSE4::URA leu2,3-112 his3-11 trp1-1 ade2-1 can1-100 bar1-1 bud4 RAD5 htz1::HYG::pHTZ1-3HA-HTZ1::TRP cse4::KAN swr1::HYG*	*pSB1067, pSB1729*
SBY12922	*MAT**a** ura3-1::pCSE4-3Flag-CSE4::URA leu2,3-112 his3-11 trp1-1 ade2-1 can1-100 bar1-1 bud4 RAD5 htz1::HYG::pHTZ1-3HA-HTZ1::TRP*	*pSB1067, pSB1072, pSB1729*

	cse4::KAN swr1::HYG psh1::KAN	
SBY12924	*MAT***a** *ura3-1::pCSE4-3Flag-CSE4::URA leu2,3-112::pGAL-3Flag-CSE4::LEU2 his3-11 trp1-1 ade2-1 can1-100 bar1-1 bud4 RAD5 htz1::HYG::pHTZ1-3HA-HTZ1::TRP cse4::KAN swr1::HYG psh1::KAN*	*pSB1067, pSB1729, pSB1072*
SBY12928	*MAT***a** *ura3-1::pCSE4-3Flag-CSE4::URA leu2,3-112 his3-11 trp1-1 ade2-1 can1-100 bar1-1 bud4 RAD5 htz1::HYG::pHTZ1-3HA-HTZ1::TRP cse4::KAN nhp10::HYG psh1::KAN*	*pSB1067*
SBY12930	*MAT***a** *ura3-1::pCSE4-3Flag-CSE4::URA leu2,3-112::pGAL-3Flag-CSE4::LEU2 his3-11 trp1-1 ade2-1 can1-100 bar1-1 bud4 RAD5 htz1::HYG::pHTZ1-3HA-HTZ1::TRP cse4::KAN nhp10::HYG*	*pSB1067, pSB1072, pSB1729*
SBY12956	*MAT***a** *ura3-1::pCSE4-3Flag-CSE4::URA leu2,3-112::pGAL-3Flag-CSE4::LEU2 his3-11 trp1-1 ade2-1 can1-100 bar1-1 bud4 RAD5 htz1::HYG::pHTZ1-3HA-HTZ1::TRP cse4::KAN swr1::HYG*	*pSB1067, pSB1072, pSB1729*
SBY12958	*MAT***a** *ura3-1::pCSE4-3Flag-CSE4::URA leu2,3-112 his3-11 trp1-1 ade2-1 can1-100 bar1-1 bud4 RAD5 htz1::HYG::pHTZ1-3HA-HTZ1::TRP*	*pSB1067, pSB1072*

	cse4::KAN nhp10::HYG	
SBY12959	MAT**a** ura3-1::pCSE4-3Flag-CSE4::URA leu2,3-112::pGAL-3Flag-CSE4::LEU2 his3-11 trp1-1 ade2-1 can1-100 bar1-1 bud4 RAD5 htz1::HYG::pHTZ1-3HA-HTZ1::TRP cse4::KAN nhp10::HYG psh1::KAN	pSB1067, pSB1072, pSB1729
SBY13702	MAT**a** ura3-1 leu2,3-112 his3-11 trp1-1 ade2-1 can1-100 bar1-1 bud4 rad5-535 htz1::HYG::pHTZ1-3HA-HTZ1::TRP	pSB1072
SBY13998	MAT**a** ura3-1::pCSE4-3Flag-CSE4::URA leu2,3-112 his3-11 trp1-1 ade2-1 can1-100 bar1-1 bud4 RAD5 htz1::HYG::pHTZ1-3HA-HTZ1::TRP cse4::KAN psh1::KAN	pSB1067, pSB1072
SBY14482	MAT**a** ura3-1::pCSE4-3Flag-CSE4::URA leu2,3-112::pGAL-3Flag-CSE4::LEU2 his3-11 trp1-1 ade2-1 can1-100 bar1-1 bud4 RAD5 cse4::KAN PSH1-13Myc::HIS htz1::HYG::pHTZ1-3HA-HTZ1::TRP	pSB1067 pSB1729
SBY14515	MAT**a** ura3-1 leu2,3-112::pGAL-3Flag-CSE4::LEU2 his3-11 trp1-1 ade2-1 can1-100 bar1-1 bud4 rad5-535 INO80-13Myc::HIS psh1::KAN	pSB1729
SBY14526	MAT**a** ura3-1 leu2,3-112::pGAL-3Flag-	pSB1729

	CSE4::LEU2 his3-11 trp1-1 ade2-1 can1-100 bar1-1 bud4 rad5-535 INO80-13Myc::HIS	
SBY14527	*MAT***a** *ura3-1 leu2,3-112 his3-11 trp1-1 ade2-1 can1-100 bar1-1 bud4 rad5-535 INO80-13Myc::HIS*	
SBY15903	*MAT***a** *ura3-1 leu2,3-112 his3-11 trp1-1 ade2-1 can1-100 bar1-1 rad5-535 2uM::pTET-CSE4::URA*	*[pSB1073]*
SBY15904	*MAT***a** *ura3-1 leu2,3-112 his3-11 trp1-1 ade2-1 can1-100 bar1-1 rad5-535 2uM::pTET-CSE4::URA psh1::KAN*	*[pSB1073]*
SBY15906	*MAT***a** *ura3-1 leu2,3-112 his3-11 trp1-1 ade2-1 can1-100 bar1-1 rad5-535 2uM::pTET-CSE4::URA psh1::KAN htz1::KAN*	*[pSB1073]*
SBY15924	*MAT***a** *ura3-1 leu2,3-112 his3-11 trp1-1 ade2-1 can1-100 bar1-1 rad5-535 2uM::pTET-Empty::URA*	*[pSB416]*
SBY16018	*MAT***a** *ura3-1 leu2,3-112 his3-11 trp1-1 ade2-1 can1-100 bar1-1 bud4 RAD5 psh1::KAN*	
SBY16066	*MAT***a** *ura3-1 leu2,3-112::pGAL-3Flag-CSE4:LEU2 his3-11 trp1-1 ade2-1 can1-100 bar1-1 bud4 RAD5*	*pSB1729*
SBY16072	*MAT***a** *ura3-1 leu2,3-112 his3-11 trp1-1 ade2-1*	

	can1-100 bar1-1 bud4 RAD5	
SBY16074	*MAT**a** ura3-1 leu2,3-112 his3-11 trp1-1 ade2-1 can1-100 bar1-1 bud4 RAD5 htz1::HYG*	
SBY16076	*MAT**a** ura3-1 leu2,3-112 his3-11 trp1-1::pGAL-H3::TRP1 ade2-1 can1-100 bar1-1 bud4 RAD5*	*pSB893*
SBY16080	*MAT**a** ura3-1 leu2,3-112 his3-11 trp1-1 ade2-1 can1-100 bar1-1 bud4 RAD5*	
SBY16082	*MAT**a** ura3-1 leu2,3-112 his3-11 trp1-1 ade2-1 can1-100 bar1-1 bud4 RAD5 htz1::HYG*	
SBY16084	*MAT**a** ura3-1 leu2,3-112 his3-11 trp1-1::pGAL-H3::TRP1 ade2-1 can1-100 bar1-1 bud4 RAD5*	*pSB893*
SBY16099	*MAT**a** ura3-1 leu2,3-112 his3-11 trp1-1 ade2-1 can1-100 bar1-1 bud4 RAD5 psh1::KAN*	
SBY16101	*MAT**a** ura3-1 leu2,3-112 his3-11 trp1-1 ade2-1 can1-100 bar1-1 bud4 RAD5 psh1::KAN*	
SBY16102	*MAT**a** ura3-1 leu2,3-112::pGAL-3Flag-CSE4::LEU2 his3-11 trp1-1 ade2-1 can1-100 bar1-1 bud4 RAD5*	*pSB1729*
SBY16103	*MAT**a** ura3-1 leu2,3-112::pGAL-3Flag-CSE4:LEU2 his3-11 trp1-1 ade2-1 can1-100 bar1-1 bud4 RAD5 psh1::HYG*	*pSB1729*

References

1. Lindstrom KC, Vary JC, Jr., Parthun MR, Delrow J, Tsukiyama T. Isw1 functions in parallel with the NuA4 and Swr1 complexes in stress-induced gene repression. Mol Cell Biol. 2006 Aug;26(16):6117-29. PubMed PMID: 16880522. PMCID: PMC1592817.
2. Vincent JA, Kwong TJ, Tsukiyama T. ATP-dependent chromatin remodeling shapes the DNA replication landscape. Nat Struct Mol Biol. 2008 May;15(5):477-84. PubMed PMID: 18408730. PMCID: PMC2678716.
3. Alcid EA, Tsukiyama T. ATP-dependent chromatin remodeling shapes the long noncoding RNA landscape. Genes Dev. 2014 Nov 1;28(21):2348-60. PubMed PMID: 25367034. PMCID: PMC4215180.
