# Supplementary material for: Regulation of Budding Yeast CENP-A levels Prevents Misincorporation at Promoter Nucleosomes and Transcriptional Defects
Source: PLoS Genet. 2016 Mar 16;12(3):e1005930. doi: 10.1371/journal.pgen.1005930 (PMC4794243; doi:10.1371/journal.pgen.1005930)
Supplement: S3 Table — (PDF) [file pgen.1005930.s012.pdf]

S3 Table. Plasmids used for this study.

Plasmid Number	Description	Source
pSB1067	*pCSE4-3Flag-CSE4* + 500bp downstream, *URA3* (integrating)	[1]
pSB1072	*pHTZ1-3HA-HTZ1* + 250bp downstream, *TRP1* (integrating)	Biggins Lab
pSB1729	*pGAL-3Flag-CSE4* + 500bp downstream, *LEU2* (integrating)	Biggins Lab
pSB893	*pGAL-HHT1, TRP1* (integrating)	[2]
pSB1073	*pTET-CSE4, URA3,* 2μM	Biggins Lab
pSB416 (CM190)	*pTET-empty vector*, URA3, 2μ*M*	[3]

References

1. Deyter GMR, Biggins S. The FACT complex interacts with the E3 ubiquitin ligase Psh1 to prevent ectopic localization of CENP-A. Genes Dev. 2014 Aug 15;28(16):1815-26.

2. Ranjitkar P, Press MO, Yi X, Baker R, MacCoss MJ, Biggins S. An E3 ubiquitin ligase prevents ectopic localization of the centromeric histone H3 variant via the centromere targeting domain. Mol Cell. 2010 Nov 12;40(3):455-64. PubMed PMID: 21070971. PMCID: 2995698. Epub 2010/11/13.

3. Gari E, Piedrafita L, Aldea M, Herrero E. A set of vectors with a tetracycline-regulatable promoter system for modulated gene expression in *Saccharomyces cerevisiae*. Yeast. 1997;13:837-48.
