# Supplementary material for: Regulation of Budding Yeast CENP-A levels Prevents Misincorporation at Promoter Nucleosomes and Transcriptional Defects
Source: PLoS Genet. 2016 Mar 16;12(3):e1005930. doi: 10.1371/journal.pgen.1005930 (PMC4794243; doi:10.1371/journal.pgen.1005930)
Supplement: S4 Table — (PDF) [file pgen.1005930.s013.pdf]

S4 Table. Oligonucleotides used for this study.

Oligo Number	Purpose	Sequence	Source
SB773	*CEN3* Forward	ATCAGCGCCAAACAATATGGAAAA	[1]
SB774	*CEN3* Reverse	GAGCAAAACTTCCACCAGTAAACG	[1]
SB3061	*CEN4* forward	TCACATGCTTATAATCAACTTTT	K. Krassovsky, S. Henikoff
SB3062	*CEN4* Reverse	TGTTTCGGTAATCATAAACAA	K. Krassovsky, S. Henikoff
SB3735	*SAP4* Promoter Forward	ACAGCACAACACGCTTACCA	This study
SB3736	*SAP4* Promoter Reverse	CCAGCCCTAAATCCCCTAAA	This study
SB3781	*SLP1* Promoter Forward	TCCTAGGTTATCTCATCGGTACT	This study
SB3782	*SLP1* Promoter Reverse	ACTATATCCATTGCGTCCTTTCT	This study
SB3814	*UTH1* Gene Forward	GTAACACCGCCACCTCTTGT	This study
SB3815	*UTH1* Gene Reverse	ACCATCGGAAGGTTGTTCAG	This study

SB4768 (CR2)	*RDS1* Promoter Forward	GACCCGTGCAGATCACTATTACA	[2]
SB4769 (CR2)	*RDS1* Promoter Reverse	GCAGTTTATCACATTTCCGTTTG	[2]
SB4983 (TT4412)	*rDNA (RDN25)* Forward	GCCTGTGGGAATACTGCCAG	L. Lee, T. Tsukiyama
SB4984 (TT4413)	*rDNA (RDN25)* Reverse	CCATCTTTCGGGTCCCAACAGC	L. Lee, T. Tsukiyama
SB4762	*ARE1* Gene	AAGGAATCTTTGTCCCCAGAGA	[2]
SB4763	*ARE1* Gene	TCTTGGGTAGTTGATCTGGTACA C	[2]

References

1. Collins KA, Castillo AR, Tatsutani SY, Biggins S. De novo kinetochore assembly requires the centromeric histone H3 variant. Mol Biol Cell. 2005;16:5649-60.
2. Mizuguchi G, Shen X, Landry J, Wu WH, Sen S, Wu C. ATP-driven exchange of histone H2AZ variant catalyzed by SWR1 chromatin remodeling complex. Science. 2004 Jan 16;303(5656):343-8. PubMed PMID: 14645854.
