# Supplementary material for: Regulation of Budding Yeast CENP-A levels Prevents Misincorporation at Promoter Nucleosomes and Transcriptional Defects
Source: PLoS Genet. 2016 Mar 16;12(3):e1005930. doi: 10.1371/journal.pgen.1005930 (PMC4794243; doi:10.1371/journal.pgen.1005930)
Supplement: S5 Table — (PDF) [file pgen.1005930.s014.pdf]

S5 Table: RNA-seq information

Sample Name	Strain number	Mapped Reads
WT t0 batch 1	SBY3939	8,052,352
psh1ΔKAN t0 batch 1	SBY16099	8,479,755
psh1ΔKAN leu2-3,112::pGAL-3Flag-CSE4:LEU2 t0 batch 1	SBY12350	7,285,227
htz1ΔHYG t0 batch 1	SBY6183	8,736,689
trp1-1::pGAL-H3:TRP1 t0 batch 1	SBY4471	8,066,430
WT t2 batch 1	SBY3939	7,748,726
psh1ΔKAN t2 batch 1	SBY16099	7,513,193
psh1ΔKAN leu2-3,112::pGAL-3Flag-CSE4:LEU2 t2 batch 1	SBY12350	7,392,085
htz1ΔHYG t2 batch 1	SBY6183	7,003,319
trp1-1::pGAL-H3:TRP1 t2 batch 1	SBY4471	7,926,433
WT t0 batch 2	SBY16072	7,652,177
psh1ΔKAN t0 batch 2	SBY16101	7,471,872
leu2-3,112::pGAL-3Flag-CSE4:LEU2 t0 batch 2	SBY16102	7,531,780
psh1ΔKAN leu2-3,112::pGAL-3Flag-CSE4:LEU2 t0 batch 2	SBY12338	6,919,807
htz1ΔHYG t0 batch 2	SBY16074	7,035,485
trp1-1::pGAL-H3:TRP1 t0 batch 2	SBY16076	6,975,987
WT t2 batch 2	SBY16072	7,227,820
psh1ΔKAN t2 batch 2	SBY16101	7,702,140
leu2-3,112::pGAL-3Flag-CSE4:LEU2 t2 batch 2	SBY16102	7,740,670
psh1ΔKAN leu2-3,112::pGAL-3Flag-CSE4:LEU2 t2 batch 2	SBY12338	6,922,124

htz1ΔHYG t2 batch 2	SBY16074	7,162,966
trp1-1::pGAL-H3:TRP1 t2 batch 2	SBY16076	7,758,302
WT t0 batch 3	SBY16080	7,239,742
psh1ΔKAN t0 batch 3	SBY16018	7,337,970
leu2-3,112::pGAL-3Flag-CSE4:LEU2 t0 batch 3	SBY16066	6,778,973
psh1ΔKAN leu2-3,112::pGAL-3Flag-CSE4:LEU2 t0 batch 3	SBY16103	7,624,807
htz1ΔHYG t0 batch 3	SBY16082	7,267,024
trp1-1::pGAL-H3:TRP1 t0 batch 3	SBY16084	7,764,848
WT t2 batch 3	SBY16080	7,854,429
psh1ΔKAN t2 batch 3	SBY16018	7,302,453
leu2-3,112::pGAL-3Flag-CSE4:LEU2 t2 batch 3	SBY16066	7,518,238
psh1ΔKAN leu2-3,112::pGAL-3Flag-CSE4:LEU2 t2 batch 3	SBY16103	7,228,229
htz1ΔHYG t2 batch 3	SBY16082	6,867,970
trp1-1::pGAL-H3:TRP1 t2 batch 3	SBY16084	7,862,726
