# Supplementary material for: Regulation of Budding Yeast CENP-A levels Prevents Misincorporation at Promoter Nucleosomes and Transcriptional Defects
Source: PLoS Genet. 2016 Mar 16;12(3):e1005930. doi: 10.1371/journal.pgen.1005930 (PMC4794243; doi:10.1371/journal.pgen.1005930)
Supplement: S3 File — (PDF) [file pgen.1005930.s017.pdf]

S3 File: Supplemental Methods

ChIP-qPCR to validate CENP-A^{Cse4} peaks

ChIP DNA samples prepared for ChIP-seq were analyzed by quantitative real-time PCR (qPCR) (7900HT, ABI Prism) to validate CENP-A^{Cse4} peaks as described [1] with the following modifications to the data analysis. A standard curve of input DNA was run for each primer set, and the replication efficiency of each primer set was calculated based on these values. The percent input was then calculated using the efficiency calibrated ΔCt method [2] for each ChIP sample taking into account the dilutions of both the input and ChIP samples for each strain. Oligo sequences are listed in S3 Table.

Comparison to CLRs and LCNCRs

CLR and LCNCR coordinates [3] were lifted over from the UCSC SacCer2 to the UCSC SacCer3 genome before comparing to the CENP-A^{Cse4} peaks from this study. The proportions of CLRs or LCNCRs that overlap CENP-A^{Cse4} ChIP-seq peaks in this study were calculated using R Bioconductor packages rtracklayer, Rsamtools, and BSgenome.Scerevisiae.UCSC.sacCer3 [4-8]. Graph showing proportions was made using GraphPad Prism version 6.0 for OSX, Graphpad Software, La Jolla California USA, www.graphpad.com.

% AT calculation

% AT/CENP-A^{Cse4} or input nucleosome peak for each strain was calculated in R using Bioconductor packages rtracklayer, Rsamtools, and BSgenome.Scerevisiae.UCSC.sacCer3 [4-8]

Boxplots of the distributions of %AT/peak were plotted using GraphPad Prism version 6.0 for OSX, Graphpad Software, La Jolla California USA, www.graphpad.com.

H2A.Z^{Htz1} score distributions

Boxplots of the distribution of the H2A.Z^{Htz1} score for either H2A.Z^{Htz1} peaks that overlap CENP-A^{Cse4} peaks or those that do not overlap CENP-A^{Cse4} peaks in all CENP-A^{Cse4} ChIP-seq strains were made using R Bioconductor packages GenomicRanges and rtracklayer [4, 6, 7] and GraphPad Prism version 6.0 for OSX, Graphpad Software, La Jolla California USA, www.graphpad.com.

CENP-A^{Cse4} stability assay

CENP-A^{Cse4} stability assays were performed as previously described [9], with the following modifications. Yeast were grown at 23 ^{o}C throughout the experiment. The (-) sample for each strain was taken before inducing *pGAL-3Flag-CSE4* overexpression. After taking the (-) sample, all strains were induced with 2% galactose for 2 hours before adding 2% glucose and 50ug/ml cyclohexamide to begin the stability assay time course.

qPCR measurement of rDNA copy number ratio

Genomic DNA was extracted using phenol:chloroform extraction with bead beating followed by ethanol precipitation [10]. The genomic DNA was RNAse A treated, phenol:chloroform extracted and then ethanol precipitated. rDNA and *UTH1* copy number were analyzed by quantitative real-time PCR (qPCR) (7900HT, ABI Prism) as described [1] with the following modifications to the data analysis. A standard curve of input DNA was run for each

primer set, and the replication efficiency of each primer set was calculated based on these values. The rDNA:*UTH1* ratio was calculated for each experimental strain relative to a WT strain (SBY3) using the efficiency calibrated ΔCt method [2]. Oligo sequences are listed in S3 Table. Graphs were made using GraphPad Prism version 6.0 for OSX, Graphpad Software, La Jolla California USA, www.graphpad.com.

qPCR measurement of rRNA transcript level

0.5 ug samples of the DNAse-treated RNA that was used for the RNA-seq experiment were made into cDNA using the iScript™ Reverse Transcription Supermix for RT-qPCR (Bio-Rad). rRNA and *ACT1* transcript levels were analyzed by quantitative real-time PCR (qPCR) (7900HT, ABI Prism) as described [1] with the following modifications to the data analysis. A standard curve of input DNA was run for each primer set, and the replication efficiency of each primer set was calculated based on these values. The rRNA:*ACT1* ratio was calculated for each experimental strain at t = 0 and t = 2 hours relative to a WT strain (SBY3) at t = 0 using the efficiency calibrated ΔCt method [2]. Oligo sequences are listed in S3 Table. Graphs were made using GraphPad Prism version 6.0 for OSX, Graphpad Software, La Jolla California USA, www.graphpad.com.

Comparison of transcriptional changes with cell cycle regulated genes

Genes significantly up- or down-regulated in the *psh1*Δ *pGAL-3Flag-CSE4* strain at t2 compared to t0 were compared to a list of cell cycle regulated genes [11] using Microsoft Excel. Histograms were made of the cell cycle % where the expression of these genes peaked using

using GraphPad Prism version 6.0 for OSX, Graphpad Software, La Jolla California USA, www.graphpad.com. 1% cell cycle = M/G1, 100% cell cycle = the next M/G1.

Comparison of transcriptional changes with mislocalized CENP-A^{Cse4} peaks

The list of genes with significant changes in transcript level from the RNA-seq analysis were compared with the list of genes with CENP-A^{Cse4} peaks within their promoters (500 bp upstream of TSS) in the *psh1*Δ *pGAL-3Flag-CSE4* strain using the Whitehead Institute Compare Two Lists tool (http://jura.wi.mit.edu/bioc/tools/compare.php). Venn diagram was drawn using BioVenn [12]. The number of significantly changed transcripts that overlapped between the strains was compared using the hypergeometric distribution (p-value is probability of getting more than the observed number of successes) using the total number of genes present in both the differential transcription and CENP-A^{Cse4} peak analysis as the total population, using the GeneProf hypergeometric distribution calculator [13].

Transcription factor enrichment analysis

Gene lists of all genes down-regulated in *psh1*Δ *pGAL-3Flag-CSE4* with promoter CENP-A^{Cse4} peaks and of all genes up-regulated in both *psh1*Δ *pGAL-3Flag-CSE4* and in *htz1*Δ were analyzed for transcription factor enrichment using YEASTRACT[14-18]. Lists of enriched transcription factors are included in S2 File.

References

1. Ng TM, Lenstra TL, Duggan N, Jiang S, Ceto S, Holstege FC, et al. Kinetochore Function and Chromosome Segregation Rely on Critical Residues in Histones H3 and H4 in Budding Yeast. Genetics. 2013;195(3):795-807.
2. Pfaffl MW. A new mathematical model for relative quantification in real-time RT-PCR. Nucleic Acids Res. 2001 May 1;29(9):e45. PubMed PMID: 11328886. PMCID: PMC55695.
3. Lefrançois P, Auerbach RK, Yellman CM, Roeder GS, Snyder M. Centromere-Like Regions in the Budding Yeast Genome. PLoS Genet. 2013 Feb 17;9(1):e1003209.
4. Gentleman RC, Carey VJ, Bates DM, Bolstad B, Dettling M, Dudoit S, et al. Bioconductor: open software development for computational biology and bioinformatics. Genome Biol. 2004;5(10):R80. PubMed PMID: 15461798. PMCID: 545600.
5. Team TBD. BSgenome.Scerevisiae.UCSC.sacCer3: *Saccharomyces cerevisiae* (Yeast) full genome (UCSC version sacCer3). R package version 1.3.1000.
6. Lawrence M, Gentleman R, Carey V. rtracklayer: an R package for interfacing with genome browsers. Bioinformatics. 2009 Jul 15;25(14):1841-2. PubMed PMID: 19468054. PMCID: 2705236.
7. Lawrence M, Huber W, Pages H, Aboyoun P, Carlson M, Gentleman R, et al. Software for computing and annotating genomic ranges. PLoS Comput Biol. 2013;9(8):e1003118. PubMed PMID: 23950696. PMCID: 3738458.
8. Morgan M PH, Obenchain V, Hayden N. . Rsamtools: Binary alignment (BAM), FASTA, variant call (BCF), and tabix file import. R package version 1.22.0 ed. Bioconductor.

9. Ranjitkar P, Press MO, Yi X, Baker R, MacCoss MJ, Biggins S. An E3 Ubiquitin Ligase Prevents Ectopic Localization of the Centromeric Histone H3 Variant via the Centromere Targeting Domain. Mol Cell. 2010 Nov 12;40(3):455-64. PubMed PMID: 21070971. PMCID: 2995698. Epub 2010/11/13.

10. Rose MD, Winston F, Heiter P. Methods in yeast genetics. Cold Spring Harbor, N. Y.: Cold Spring Harbor Laboratory Press; 1990. p. 198 p.

11. Granovskaia MV, Jensen LJ, Ritchie ME, Toedling J, Ning Y, Bork P, et al. High-resolution transcription atlas of the mitotic cell cycle in budding yeast. Genome Biol. 2010;11(3):R24. PubMed PMID: 20193063. PMCID: PMC2864564.

12. Hulsen T, de Vlieg J, Alkema W. BioVenn - a web application for the comparison and visualization of biological lists using area-proportional Venn diagrams. BMC Genomics. 2008;9:488. PubMed PMID: 18925949. PMCID: PMC2584113.

13. Halbritter F, Vaidya HJ, Tomlinson SR. GeneProf: analysis of high-throughput sequencing experiments. Nat Methods. 2012 Jan;9(1):7-8. PubMed PMID: 22205509.

14. Teixeira MC, Monteiro P, Jain P, Tenreiro S, Fernandes AR, Mira NP, et al. The YEASTRACT database: a tool for the analysis of transcription regulatory associations in *Saccharomyces cerevisiae*. Nucleic Acids Res. 2006 Jan 1;34(Database issue):D446-51. PubMed PMID: 16381908. PMCID: PMC1347376.

15. Monteiro PT, Mendes ND, Teixeira MC, d'Orey S, Tenreiro S, Mira NP, et al. YEASTRACT-DISCOVERER: new tools to improve the analysis of transcriptional regulatory associations in *Saccharomyces cerevisiae*. Nucleic Acids Res. 2008 Jan;36(Database issue):D132-6. PubMed PMID: 18032429. PMCID: PMC2238916.

16. Abdulrehman D, Monteiro PT, Teixeira MC, Mira NP, Lourenco AB, dos Santos SC, et al. YEASTRACT: providing a programmatic access to curated transcriptional regulatory associations in *Saccharomyces cerevisiae* through a web services interface. Nucleic Acids Res. 2011 Jan;39(Database issue):D136-40. PubMed PMID: 20972212. PMCID: PMC3013800.

17. Teixeira MC, Monteiro PT, Guerreiro JF, Goncalves JP, Mira NP, dos Santos SC, et al. The YEASTRACT database: an upgraded information system for the analysis of gene and genomic transcription regulation in *Saccharomyces cerevisiae*. Nucleic Acids Res. 2014 Jan;42(Database issue):D161-6. PubMed PMID: 24170807. PMCID: PMC3965121.

18. Teixeira MC, Monteiro PT, Sa-Correia I. Predicting Gene and Genomic Regulation in *Saccharomyces cerevisiae*, using the YEASTRACT Database: A Step-by-Step Guided Analysis. Methods Mol Biol. 2016;1361:391-404. PubMed PMID: 26483034.
